# Nitrogen legacies in anthropogenic landscapes: a case study in the Mondego Basin in Portugal

**DOI:** 10.1007/s11356-021-16725-x

**Published:** 2021-11-24

**Authors:** João Marques, Joy Liu, Maria C. Cunha, Kimberly J. Van Meter, Nandita B. Basu

**Affiliations:** 1 University of Coimbra, CEMMPRE, Department of Civil Engineering, University of Coimbra, Polo 2, 3030-788 Coimbra, Portugal; 2 Department of Earth and Environmental Sciences, University of Waterloo, Waterloo, Ontario N2L 3G1 Canada; 3 Department of Earth and Environmental Sciences, University of Illinois at Chicago, Chicago, IL 60607 USA; 4 Department of Civil and Environmental Engineering, University of Waterloo, Waterloo, Ontario N2L 3G1 Canada

**Keywords:** Anthropogenic nitrogen loads, Agricultural management practices, Fertilization, Nitrogen fluxes, Legacies, Water quality

## Abstract

Nitrogen (N) legacies have built up in anthropogenic landscapes over decades of agricultural intensification, and these legacies lead to time lags in water quality change measurable even beyond the moment of application of N. It is important to understand these legacies to quantify the relationship between N inputs and N concentrations in streams and implement best management practices for water quality improvement; however, little is known about the magnitude of legacies in various landscape elements like soils and groundwater. Here, we have used the ELEMeNT (Exploration of Long-tErM Nutrient Trajectories) model to explore the buildup and depletion of N legacies over a 216-year period, across the Mondego River Basin, a 6645-km^2^ watershed in Portugal, where human interventions have considerably changed the characteristics of the basin to prevent floods and improve farming conditions in recent decades. The results show that the increase in the amount of inorganic fertilizer applied was the main driver for the anthropogenic N loads in the watershed from 1950 until the beginning of the 1990s. The N inputs have been decreasing since then, but N loads in the river did not document any decrease till the 1990s; after which there was a decline. This time lag between the N inputs to the watershed and the N loads in the river (about two decades) is a function of accumulation of N legacy.

**Supplementary Information:**

The online version contains supplementary material available at 10.1007/s11356-021-16725-x.

## Introduction

Nitrogen (N) is one of the most common chemical elements and is essential for all life forms on our planet. The natural nitrogen cycle includes the conversion of N gas from the atmosphere to ammonia and nitrates by mainly lightning and biological fixation processes. These reactive N ammonia and nitrates are taken up by plants and when animals consume the plants, they host these reactive N sources. When plants and animals decompose, the incorporated N is released into the soil as ammonia, which is converted to nitrates via nitrifying bacteria. Finally, these nitrates are converted to N gas again by denitrification and released into the atmosphere, thus closing the N cycle (Zhang 2016). Human actions have altered this natural nitrogen cycle since the industrial revolution (Han et al. 2020). The creation of reactive N by human activities has exceeded the natural capacity of N fixation (Zhang 2016). These activities include burning fossil fuels that increased N atmospheric pollution, applying inorganic fertilizers in agriculture to increase crop production, and cultivating leguminous crops with high rates of N fixation. The past century has seen a massive increase in reactive N across the globe which transformed the N cycle and caused environmental damage including ozone depletion, greenhouse effect, acid rain, nitrate pollution of water bodies, eutrophication, and creation of coastal hypoxic zones. Midolo et al. (2019) claim that the N contamination of water bodies could be a serious health threat to humans and animals since an overload of N deposit in soils can destroy plants and reduce the diversity of ecosystems. Pennino et al. (2020) identify nitrates as one of the main contaminants of public drinking water systems in the USA in the last two decades.

The anthropogenic induced N input has been quantified by several authors by means of a methodology called net anthropogenic nitrogen inputs (NANIs), proposed by Howarth et al. (1996) and applied in numerous studies such as in Hong et al. (2013), Gao et al. (2015), Dupas et al. (2016), Zhang (2016), and Zhang et al. (2020). Furthermore, Boyer et al. (2002) and Hong et al. (2013) show that NANIs can be employed to estimate the N exported by rivers. However, according to Van Meter et al. (2017), NANIs are computed for “snapshots in time or multiyear averaging,” which limits the capacity of this approach to explain long-term changes in N inputs. There are important time lags between the N inputs and the measurable effects in the riverine outlet, and this has been recognized by several authors (Sanford and Pope 2013; Van Meter and Basu 2015; Ilampooranan et al. 2019).

These legacies are mentioned in Van Meter et al. (2017) as the “N which remains within a watershed at least 1 year beyond its initial application at the land surface” and could range from a single year to decades or even centuries and remain as an important N input contributor to water bodies. The work of Grimvall et al. (2000) claims that no reduction of the riverine N export of the eastern rivers occurs, despite the reduction in fertilization across European countries since the 1990s. This is due to time lags, and Van Meter and Basu (2017), for instance, estimate they are of an order of magnitude of 26 years for the Grand River Watershed in Canada.

The European Union has approved the Water Framework Directive (WFD, 2020) and the European Community Nitrates Directive (Council Directive (1991), 1991/676/EEC) to improve and control the water quality, such as by reducing the N inputs to bodies of water. This is the main legislation defining the best managements practices (BMPs) to reduce the concentration of nitrates in water bodies. But, as already mentioned, these BMPs could have a delayed impact on water quality.

To understand the efficacy of measures foreseen in the legislation already mentioned, it is necessary to quantify legacies. In this work, the ELEMeNT (Exploration of Long-tErM Nutrient Trajectories) model developed by Van Meter et al. (2017) will be explored in detail to analyze these effects. The features of ELEMeNT used to track the legacies resulting from past and present agriculture sector practices are essential to developing new future management interventions. This framework was applied to North American watersheds in Van Meter and Basu (2015) and Van Meter et al. (2017, 2018) and the results show the importance of considering these legacies.

An area in the central region of Portugal, the Mondego basin, has been studied to understand and evaluate long-term nutrient trajectories between 1800 and 2016 that are used to quantify the legacies. The Mondego watershed is an interesting setting in which to apply the ELEMeNT approach. In fact, it has been considerably altered by human interventions in the last 50 years to prevent floods and to improve the agricultural conditions in a low area of the basin. These interventions featured the construction of dams on the upper Mondego to control floods and action to improve farming conditions in the lower part. These improvements followed a plan (*Projecto de Desenvolvimento Agrícola do Baixo Mondego* (DGHEA 1983)) that involved constructing new irrigation, drainage, and road infrastructure and implementing land consolidation programs. This plan was designed in the early 1980s, but it still has not been completed.

The use of methodologies to quantify N legacies is a relatively recent area of research. The ELEMeNT has been successfully applied to intensive agriculture North American watersheds with very different characteristics from those of a Mediterranean watershed. This is important to understand the model’s capability to be successfully applied to different conditions. According to Van Meter et al. (2017) substantial N legacies were built up over the years in soil and groundwater in the Mississippi and Susquehanna basins and in Grand River watershed in Canada according to Liu (2020). Nowadays, these have water quality problems at river outlets and are strongly impacted by N legacy sources. The Mondego is not an agriculture-dominated watershed as the North American ones that were studied and there are no water quality problems, namely in terms of nitrates. However, this does not mean that are no legacy effects of N accumulated over the years of agricultural activities. ELEMeNT can be valuable in helping to understand whether N legacies are taking place in the Mondego watershed. It is essential to understand if this model is able to capture legacies in conditions so different from those to which it has been applied.

This work seeks to answer the following research questions: How have the N inputs in the Mondego watershed changed over the past two centuries? How did these N inputs impact the variation in N accumulation/depletion in soil and in groundwater bodies? What is the magnitude of the estimated time lags for the Mondego? The way in which the ELEMeNT model addressed these different issues is fully discussed in this paper. The time lags of the Mondego River watershed are determined, as are the effects of the N inputs and outputs that have changed in the last two centuries, along with the accumulation or depletion of N in the soil and the change in water quality.

## Method and materials

### Model description

The degradation of water bodies from processes such as eutrophication are prevented by imposing corrective measures to avoid nutrient overloading from human activities like the non-point application of fertilizers in agriculture. The BMPs can be targeted to reduce the nutrient loss from the cropland to the bodies of water.

The nitrogen build-up in the watershed is either released into the water streams or accumulates in the soil and groundwater reservoirs over a period. This justifies the use of a framework that considers the time lags associated with nitrogen legacy accumulated over years of fertilization of farmland. According to Van Meter and Basu (2015), these legacies can be conceptualized as hydrologic and biochemical. Hydrologic legacies are associated with the dissolved solute (nitrogen in this case) that is slowly transported to the outlet stream by groundwater pathways and the biochemical one that is retained in the soil by the biochemical transformation of the solute. N can be accumulated in an organic form in the soil and then mineralized over time and released into the groundwater. This means that the quantification of the biochemical legacy is a function of the N accumulated in the soil and the organic N mineralization rates. Hydrological legacies are associated with the groundwater travel time distributions throughout the watershed, which are a function of climate conditions, geological factors, soil types, and other physical characteristics.

To interpret the long-term nutrient trajectories and quantify the N legacy stored in the watershed, Van Meter et al. (2017) have proposed a parsimonious process-based model, the ELEMeNT model.

**Fig. 1:**
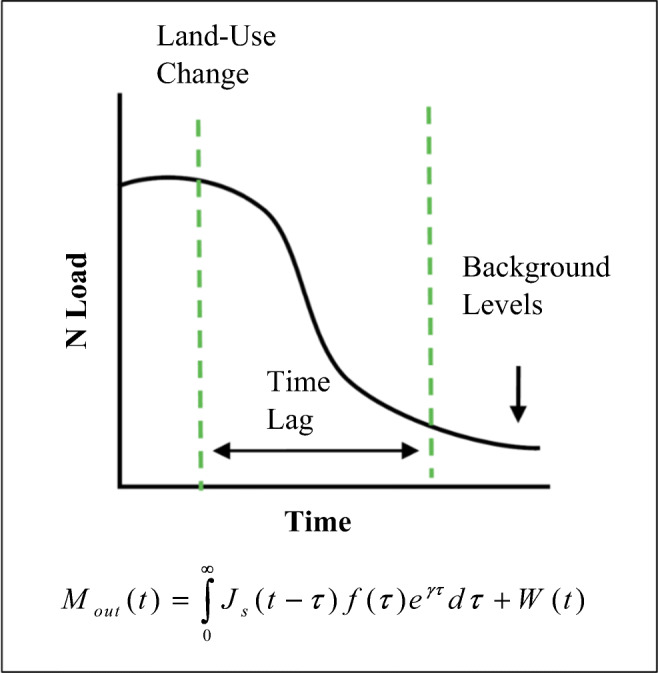
Nitrogen loading trajectories at the watershed outlet (adapted from Van Meter et al. 2017)

ELEMeNT considers source zone dynamics to model the accumulation and depletion of the soil organic nitrogen (SON) in the unsaturated soil zone and links these dynamics with the mean groundwater travel time distribution of the watershed to model the transformation and transport of the N until it reaches the watershed outlet (Fig. 1).

The watershed is divided into parts with different land use trajectories over time. The temporal evolution of each part is stored in terms of the variation of the percentage of cropland, pastureland, and non-agricultural land. For each of the three land use types, an N balance is computed based on the contribution of agricultural crops, livestock, and atmospheric deposition. The N inputs that enter the soil can be accumulated or depleted in the source zone. Van Meter et al. (2017) detail the N dynamics modeled for different land use trajectories. To sum up, the N inputs that enter the source zone are modeled by biochemical processes by dividing the zone into an active N pool (more metabolic) and a protected pool (more stable). The subdivision of the N surplus that enters the active and passive pools is determined by ELEMeNT and it is associated with the land use and tillage processes. From the biochemical transformations in the active and passive pools, the SON is mineralized into inorganic N, leaves the organic pools, and enters a mineral N pool. The mass in the mineral pool is quantified by a balance between the inputs from the SON pools and the outputs associated with denitrification processes and leaching into groundwater.

The mass leaching from the source zone into groundwater convoluted with the groundwater travel time ultimately contributes to determine the concentrations at the watershed outlet. In fact, ELEMeNT conceptualizes each portion of the area of the river basin as a stream tube with a specific travel time to the stream water line, that together lead to the groundwater travel time distribution for the watershed outlet *f*(*τ*), and then the N load at the outlet can be obtained by Eq. (1). In this equation, an additional term is considered for the contribution of the human waste to the mass of nitrates. The convolution integral conceptually means that the output value (nitrate–N in this case) represents the present and the past input values.

1 $$ {M}_{\mathrm{out}}(t)=\underset{0}{\overset{\infty }{\int }}{J}_s\left(t-\tau \right)f\left(\tau \right){e}^{\gamma \tau} d\tau +\left(1-{k}_h\right)W(t) $$

where *M*_out_(*t*) = nitrate–N mass flux at time *t*; *J*_*s*_(*t−τ*) = input of contaminant; *f*(*τ*) = travel times to the outlet stream; *γ* = first-order rate of denitrification; *k*_*h*_ = denitrification rate of human waste; and *W*(*t*) *=* human waste.

Briefly, in Eq. 1, the nitrate–N mass fluxes that reach the outlet are given as a function of the contaminant input *J*_*s*_(*t−τ*), of the travel times to the watershed outlet *f*(*τ*), and of the first-order rate of denitrification along the hydrological pathways (*γ*). The human waste *W*(*t*) is added to the mass of nitrates that reach the outlet section of the watershed. *k*_*h*_ represents the N removal from wastewater in the wastewater treatment plants and in the stream channels.

In Eq. (1), the function *J*_*s*_(*t−τ*) involves determining the N surplus (the net input of contaminant) over time in the watershed. The N surplus is obtained using a surface N balance that considers inputs and outputs from three different land uses, namely cropland (Eq. 2), pastureland (Eq. 3), and non-agricultural land (Eq. 4):

2 $$ {\mathrm{Ns}}_{\mathrm{a}}={\mathrm{BNF}}_{\mathrm{a}}+{\mathrm{FERT}}_{\mathrm{a}}+{\mathrm{MAN}}_{\mathrm{a}}+\mathrm{DEP}-\mathrm{CULT} $$

3 $$ {\mathrm{Ns}}_{\mathrm{p}}={\mathrm{BNF}}_{\mathrm{p}}+{\mathrm{FERT}}_{\mathrm{p}}+{\mathrm{MAN}}_{\mathrm{p}}+\mathrm{DEP}-\mathrm{PAST} $$

4 $$ {\mathrm{Ns}}_{\mathrm{o}}={\mathrm{BNF}}_{\mathrm{o}}+\mathrm{DEP} $$

where Ns_a_ = N surplus in cropland; BNF_a_ = biological fixation of atmospheric N in the cropland; FERT_a_ = inorganic fertilizer applied to cropland; MAN_a_ = manure applied to the cropland; DEP_a_ = atmospheric deposition at cropland; CULT = N incorporated in crops harvested; Ns_p_ = surplus in pastureland; BNF_p_ = biological fixation of atmospheric N in the pastureland FERT_p_ = inorganic fertilizer applied to pastureland; MAN_p_ = manure applied to the pastureland; DEP_p_ = atmospheric deposition at pastureland; PAST = grazing by animals; Ns_o_ = surplus in non-agricultural land; BNF_o_ = biological fixation of atmospheric N in the non-agricultural land; and DEP_o_ = atmospheric deposition in the non-agricultural land.

Ns_a_ is given by the sum of the biological fixation of atmospheric N in the cropland, the inorganic and organic fertilizations, the atmospheric deposition, and subtracting the harvested N from crops. Ns_p_ is also obtained as the sum of the biological fixation of N, inorganic and organic fertilization, the atmospheric deposition of N, and subtracting the grass from grazing. The Ns_o_ term is given by the sum of the biological fixation of N with the atmospheric deposition. The sum of the surplus of the agricultural areas, with pastureland and the non-agricultural land, and also the contribution of the wastewater produced by the residents in the watershed area represents the total N surplus of the basin.

### Model calibration

Calibrating ELEMeNT is a complex and challenging task given the different processes embraced and their interconnections, and it includes having to find the values for the parameters (presented in the case study application) that most influence the results. The calibration process follows the methodology used by Van Meter et al. (2017) and is organized in 3 stages: estimating the range of parameters, sensitivity analysis, and calibration.

#### Parameter range

The definition of reasonable intervals of parameter variation is required due to the associated uncertainty in the application of this kind of model. In ELEMeNT, a set of 10 parameters are considered: the nitrogen content in the soil in pristine conditions (*M*_s_); soil porosity (*n*); soil water content (*s*); soil mineralization rate constant of the active pool (*k*_a_); soil denitrification rate constant (*λ*_s_); protection coefficient of cultivated land (*h*_c_); protection coefficient of non-cultivated land (*h*_nc_); mean groundwater travel time (*μ*); groundwater denitrification rate constant (*γ*); and the human denitrification rate constant (*k*_pop_). These parameters are explored in the “ELEMeNT calibration results” section and for a deeper understanding, see Van Meter et al. (2017). Preferably, the ranges for the parameters should be defined from databases of the area under study, but there is generally no data for most parameters. In the case of the Mondego watershed, the first three parameter ranges(*M*_s_, *n*, and *s*) were defined with the data available in the LUCAS soil database (Orgiazzi et al. 2018). The other parameter ranges were based on a previous application of ELEMeNT (Van Meter et al., 2017) that recommend values based on the literature. The parameter ranges will be used to perform the sensitivity analysis. According to Liu (2020), these ranges should be reduced to the most sensitive parameters.

#### Sensitivity analysis

A sensitivity analysis was performed to identify the parameters with the most effect on the results, more specifically, on the outlet N loading. Sets of parameters were generated using the Latin hypercube sampling technique, which is a form of stratified Monte Carlo sampling. One thousand different sets of parameters were generated randomly considering a uniform distribution over the parameter range (Van Meter et al., 2017). With these 1000 sets of parameters, the model was run to obtain the residual sum of square values of the mean annual N loading. These results were rank-transformed to consider the non-linearities of the model (Van Meter et al., 2017). Finally, the input data (the set of randomly generated parameters) and the outputs (mean annual stream N loading) were analyzed with a stepwise regression analysis to determine which parameters have the most influence on the model results and those that can be fixed because they have minor effect on the results.

#### Parameter calibration

The purpose of the ELEMeNT calibration is to determine the best values of the parameters that lead to the results of the simulation that best fit the N load recorded at the watershed outlet. The observed N loading is based on nitrate concentrations data and discharge flows over a period of time. OSTRICH (Matott 2017) is a multi-objective optimization tool that was implemented for model calibration and makes use of a Pareto-archived dynamically dimensioned search (PA-DDS) algorithm to find acceptable solutions (Liu 2020). This PA-DDS algorithm generates sets of parameter solutions within the parameter ranges and tries to focus the search on the most relevant parameters (determined by the sensitivity analysis). Each iteration of the PA-DDS algorithm generates candidate solutions based on the best non-dominated solutions found so far, and if the newly generated solution dominates or is non-dominated by the best solutions found up to that point, the new solution is saved; otherwise, it is rejected and another candidate solution is generated from the non-dominated set. The best set of parameters is selected from the results according to the best fit of the results evaluated by the Kling–Gupta efficiency (KGE) metric proposed by Gupta et al. (2009) and computed according to Eq. 5:

5 $$ \mathrm{KGE}=1-\sqrt{{\left(\mathrm{PC}-1\right)}^2+{\left(\frac{{\mathrm{DP}}_{\mathrm{s}}}{{\mathrm{DP}}_{\mathrm{o}}}+1\right)}^2+{\left(\frac{m_s}{m_o}+1\right)}^2} $$

where PC = the Pearson correlation coefficient between the time series of the simulated and observed N loading; DP_*s*_ = the standard deviation of the simulated N loading time series; DP_*o*_ = the standard deviation of the observed N loading time series; *m*_s_ = mean of the simulated N loading time series; and *m*_o_ = mean of the observed N loading time series.

The KGE metric has been used in calibration processes of hydrological models as an objective function. This is an improved measure in relation to the most widely used Nash–Sutcliffe efficiency (NSE) metric (Nash and Sutcliffe, 1970), which is based only on the mean squared error between the series of modeled and observed values. The improvement of the KGE metric is related to the consideration of three different types of model errors related to the mean, variability, and dynamics. This measure is appropriate for use in multi-objective optimization models for calibration as it avoids an “overfitting of model parameters” for a particular aspect (Pool et al., 2018).

The calibrated values for each parameter obtained by OSTRICH are those that minimize the difference between the simulated and the observed stream N loading between the set of possibilities generated. These are the sets of parameters that lead to the highest values for the KGE metric.

### Mondego watershed general characterization

ELEMeNT model’s capabilities will be fully used to help to understand the effects related to the anthropogenic changes and to quantify the N legacy in the Mondego watershed. ELEMeNT considers past land use trajectories. This means that the model does not just consider the current land use, but it also takes into consideration the specific point in time when there was a land use change, e.g., from non-agricultural to agricultural. The next sections present the study area, its context in terms of interventions that occurred or were delayed in the past decades, and data input to the application of ELEMeNT. The European Community Nitrates Directive was transposed to each of the European countries’ national law. In the case of Portugal, this was done through a 1997 law (DL 235/97) updated by (DL 68/99).

#### Study area

**Fig. 2:**
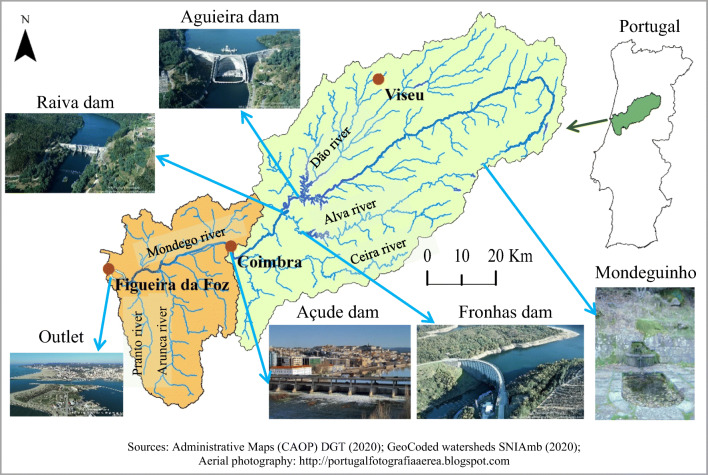
Mondego River Basin divided into the upper and lower parts and important sections of the stream network. *Sources*: Administrative Maps (CAOP) DGT (2020); GeoCoded watersheds SNIAmb (2020); Aerial photography: http://portugalfotografiaaerea.blogspot.com

The Mondego watershed (Fig. 2) has an area of 6645 km^2^ and is located in the central region of Portugal. It is bordered by the watershed of the Douro to the north, Vouga to the east, and the Tagus and Lis to the south. The Mondego River rises in the Serra da Estrela at an altitude of 1525 m (the *Mondeguinho* spring) and flows for 258 km until reaching the sea at Figueira da Foz. Its main tributaries are the Dão, Alva, Ceira, and Arunca rivers. In terms of administrative areas, the Mondego flows through the districts of Guarda, Viseu, and Coimbra.

The river starts its course within narrow valleys (over 50 km), forming rapids and waterfalls with high water velocity and turbulence. Then it crosses an upland, mainly in the Viseu and Guarda districts and receives one of its main tributaries, the River Dão. After the confluence of the Dão River, the Aguiera and Raiva dams were built on the river Mondego and the Fronhas dam on the Alva River (another major tributary of the Mondego). Then it reaches Coimbra and the Açude dam. Downstream of this point, the river flows slowly within very gentle slopes. This is the low Mondego part of the watershed and is shown in orange in Fig. 2. The light green area is the upper Mondego. Finally, it reaches the Atlantic Ocean at Figueira da Foz, after the confluence of two other important tributaries (river Arunca and river Pranto).

In the past, frequent floods in the low Mondego area used to destroy infrastructure and crops. The Mondego Watershed Hydraulic Master Plan (*Aproveitamento Hidráulico da Bacia do Mondego: plano geral* DGSH (1962)) was drafted in the 1960s to prevent floods, generate electricity, and regularize the river flow in the low Mondego. The plan started to be implemented by 1972 with the construction of the Aguieira, Raiva, Fronhas, and Acude dams, which were inaugurated in 1981 in the upper part. In the low part, the Mondego riverbanks were regularized to prevent flooding of the farmland near the water course.

**Fig. 3:**
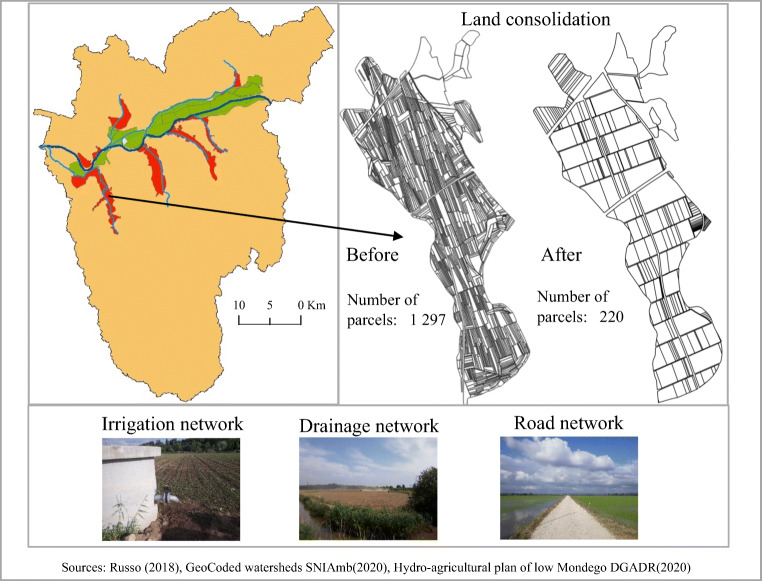
Lower sub-basin of Mondego and the blocks of agricultural land (top right), land consolidation (top left), and interventions to improve agricultural conditions (bottom). *Sources*: Russo (2018), GeoCoded watersheds SNIAmb (2020), Hydro-agricultural plan of low Mondego DGADR (2020)

The low Mondego includes an alluvial plain near the banks of the Mondego and its main tributaries with very fertile land, which is very good for agriculture. In the past, this land was underused because of the frequent floods. But once the dams have been built in the upper Mondego in 1981, the hydrological regime of the river was controlled. The Project for Agricultural Development in the Lower Mondego River Valley (*Projecto de Desenvolvimento Agrícola do Baixo Mondego* DGHEA (1983)) was developed to create conditions for farming in the lower-lying lands. Downstream of the Açude dam in Coimbra, the river was regularized to prevent flooding of the farmland and to improve the agricultural conditions by replacing traditional farming with a more specialized and intensified agricultural production. The valley was organized in blocks of land with a total area of 12,200 ha, as shown in the top right of Fig. 3.

The blocks were defined so as to secure some uniformity in terms of the physical characteristics such as soil, groundwater level and quality, topography, and existing community borders (Cunha et al. 2021). The blocks in green (Fig. 3) represent those that have already undergone intervention and those in red are still to be concluded. The interventions to improve the agricultural conditions in the blocks involve the construction of irrigation, drainage, and road networks, as well as implementing programs to increase and organize the land parcels through land consolidation programs. Some examples of these interventions are given in Fig. 3, including interventions already shown as pictures of irrigation, drainage, and road networks at the bottom of the figure and a study for land consolidation (on the top left side of the figure) in the Pranto area. The intention there is to reduce the number of land parcels from 1297 to 220 within a total area of just 323 ha. Before the implementation of the agricultural plan (DGHEA 1983), the farming practiced was undeveloped with little mechanization and using animals as a work force. The interventions were proposed for an area of 12,200 ha. Those in green represent 6706 ha that were improved between 1990 and 2015 (about 55% of the total area) and there are still 5494 ha to be improved (45%). This has consequences in terms of the abandonment of agricultural land that are further discussed below.

#### Input data for computing N surplus

To compute the N surplus using the N balance approach of Eqs. 2 to 4 for different land uses, data are required to assess the inputs of organic and inorganic fertilizers, human waste, biological nitrogen fixation, and atmospheric deposition and the outputs of crop harvesting and pastures from grazing animals.

##### Organic and inorganic N fertilization

Organic fertilization is a function of the number of animals of different species and the coefficients for N uptake and excretion given by the Code of Good Agricultural Practices (*Código de Boas Práticas Agrícolas*) (CBPA 2018) approved in DL 25/2018, 2 February, and by Hong et al. (2012). The livestock numbers are available from the INE (Statistics Portugal) (INE 2020). The evolution of the livestock of different species has changed in different ways during the period of 82 years, from 1934 to 2016. For more information, please consult the Supplementary Materials in Text S1 and Fig. S1.

**Fig. 4:**
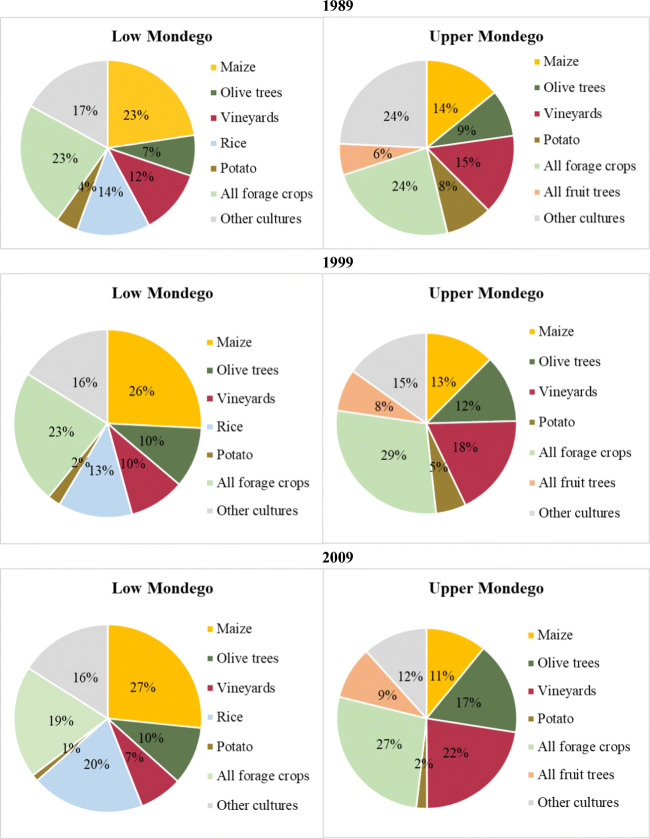
Percentages of the most important crops in the low and upper parts of Mondego watershed

The inorganic fertilization is function of the areas under cultivation and of the amount of fertilization use. The crop areas of the upper and lower Mondego parts for periods 1989 to 2016 can be consulted in Supplementary Material Table S1. The percentages of the most important crops in the low and upper Mondego are represented in Fig. 4.

The most important cultures in the low Mondego are maize and rice, mainly grown in the agricultural blocks shown in Fig. 3, and vineyards and olive trees in areas farther away from the main river course. The relative importance of maize in the low Mondego has increased from 23 to 27% (Fig. 4) and rice cultivation from 14 to 20%. In fact, maize and rice production represented almost half of the cultivated areas in the low Mondego, mostly concentrated in the agricultural blocks (shown in Fig. 3). The Project for Agricultural Development in the Lower Mondego River Valley (*Projecto de Desenvolvimento Agrícola do Baixo Mondego* DGHEA (1983)) was the driver for the development of these crops since it created conditions for farming in the lower-lying lands. In terms of the upper Mondego, the most important crops are maize, olives and vineyards. In 2016, these cultures accounted for 50% of the cultivated area. The importance of maize has decreased from 14% in 1989 to 11% in 2009 and other crops such as olives and vineyards have increased their coverage from 9 to 17% and 15 to 22%, respectively. In fact, there are important demarcated wine regions like Dão and Bairrada in this region, with large estates given over solely to vines.

The application of chemical fertilizers has been considered after 1950. Carmo et al. (2017) argue that it was in the 1950s in Portugal when there was a shift from agriculture that used only organic fertilizers such as livestock excretion to one that used an inorganic fertilizer and that this deficit greatly influenced this shift. Data about the amounts of fertilizer can be consulted in Supplementary Material Table S2 and were obtained directly from the EC (European Commission), at the Joint Research Centre of the European Union directory (https://water.jrc.ec.europa.eu/) based on the Common Agricultural Policy Regionalised Impact (CAPRI) model (Britz and Witzke 2014) and the Corine Land Cover (CLC) (Büttner 2014). The inorganic fertilization reaches a maximum in the early 1990s. The implementation of BMPs in the agricultural sector triggered a decline of fertilization use since then.

##### Residents

The ELEMeNT model considers an input N component from the contribution of human waste. This is computed as the number of residents in the Mondego watershed multiplied by a human N coefficient based on the value reported by Hong et al. (2012). In Portugal, the first INE Census was in 1864, the second in 1878, and the third in 1890. Since then, the Census has been held every 10 years, with the last one in 2011. The data from 2016 are given by the INE Population Estimates (INE 2020). The number of residents is presented in Supplementary Materials (Fig. S2) for the low and upper Mondego.

The number of residents in the low Mondego increased from 1878 until 2001 and then stayed much the same until 2016. In the upper part, the residents increase until 1950 and then the figure is approximately constant with around 420,000 residents until 2016. The growth in the number of residents between 1864 and 2016 was higher in the low Mondego—it doubled from 125,000 to 250,000—than in the upper Mondego, where it rose 60% (from 260,000 to 415,000).

##### Atmospheric N deposition

The atmospheric deposition is deemed to be the same throughout the Mondego basin and is based on the historical estimations from Deneter (2006) for long-term past values and is given by the N deposition coefficients from the European Monitoring and Evaluation Programme (EMEP) (www.emep.int) for the recent years. The N input from atmospheric deposition increased from the first decades under analysis until the end of the second millennium in 1997 to a maximum value of 6.2 kg N/ha/year and then decreased until 2016 to a value of 3.9 kg N/ha/year, associated with the decrease of N pollutant emissions into the atmosphere (Supplementary Materials (Fig. S3)).

##### Crops and pastures and biological N fixation

**Fig. 5:**
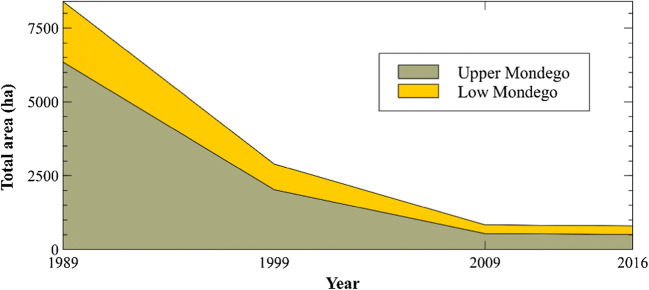
Variation of the cultivated area of crops with the capacity of biological fixation of N

The remaining data collected to compute the N surplus are the N inputs related to the capacity of the biological fixation of N and the outputs related to the removal of N by crop harvesting and by grazing animals. These terms are related to the crop and pasture areas of the Mondego watershed. The change in the cultivated area with the capacity of N fixation are presented in Fig. 5 and are based on the INE National Agricultural Census (INE-NAC) and INE Agricultural Statistics (INE-AS) (INE 2020) databases. The crop-producing areas with the biological fixation capacity have been decreasing since 1989 based on the INE-NAC and INE-AS (INE 2020) databases. For additional information, please consult the Supplementary Materials in Text S2.

The most important crops in the low and upper Mondego are represented in Fig. 4 for the years 1989, 1999, and 2009 of the INE-NAC published by INE (INE 2020) and the N incorporated in the different crops is based on the works of Hong et al. (2012) and Bouwman et al. (2005). More detailed information of the crop areas can be found in the Supplementary Materials in Text S3 and Table S1.

#### Preliminary analysis of N inputs in the Mondego watershed

**Fig. 6:**
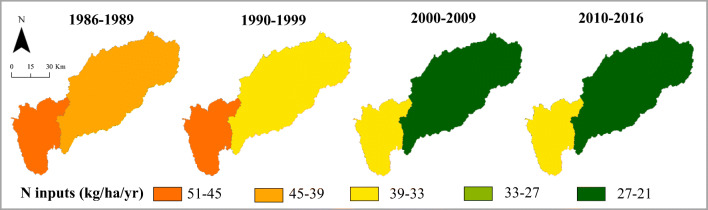
N inputs for the Mondego watershed between 1986 and 2016

There are more data sources available for the most recent decades (since the 1990s), and this allows us to make a preliminary analysis by computing the N inputs in the Mondego basin. These inputs are computed by the sum of the nitrogen that comes from the atmospheric deposition, the nitrogen contained in organic and inorganic fertilization, human waste, and biological nitrogen fixation. Data used to compute these components were described in the previous sections. The calculated inputs correspond to mean values for the periods: 1986–1989, 1990–1999, 2000–2009, and 2010–2016. The results are presented for the low and upper areas of the Mondego basin in Fig. 6.

In general, the N input values have been declining over the periods both for the upper and lower parts. The N inputs in low Mondego of the first period (1986–1989) decrease from values in a range from (51–45) kg N/ha/year to values of (39–33) kg N/ha/year in the last period (2010–2016). The same trajectory was observed in the upper Mondego with the N inputs of the first period (1986–1989) decreasing from values in a range from (45–39) kg N/ha/year to values of (27–21) kg N/ha/year in the last period (2010–2016).

This is mainly related to the decline in farming activities in the watershed and the implementation of best management practices. These practices were implemented in the agricultural sector to restrict the application of organic and inorganic fertilizers not only in terms of quantity, but also the months where the land can be fertilized. These are based on European and Portuguese legislation and the latest version is CBPA (2018).

Some abandonment of the agricultural activities was noticed particularly after the “Carnation” Revolution in 1974. In fact, the social structure of the population started to change, and residents previously engaged in the primary sector moved to other, new, activities in the urban areas (Cunha et al. 2021). There were no younger farmers to replace the old ones who had given up. Additionally, the interventions foreseen to improve the low Mondego agricultural conditions were, and still are, taking too long to be implemented (45% of the planned area is still to be improved) and this is also contributing to the abandonment of agriculture.

In any case, the N inputs from the lower part are higher than those from the upper part across all periods. These two parts of the basin are quite distinct. In the lower part, the population density (in 2016, 146 residents per km^2^) is higher than in the upper part (84 residents per km^2^ in 2016). Also, in terms of agriculture, the percentage of crop land in the lower part (16% in 2016) is higher than percentage in the upper part (14%). These differences explain the different input levels in these parts. The N inputs will be analyzed in greater detail in the next section.

#### N loads in the stream

The stream flow data were available from the Portuguese environmental agency (https://sniamb.apambiente.pt/) and were used to obtain the mean annual discharge in (m^3^/s) for a horizon from 1956 to 2016. The concentration data were also available from the Portuguese environmental agency (https://sniamb.apambiente.pt/) in terms of nitrates (mg NO_3_/L) and for a much smaller number of years from 1989 to 2015. Over this horizon, in more than 80% of the years, the water quality analysis sets available per year include six or more samples at monitoring points located at the outlet of the Mondego River. These nitrate concentration data were converted to nitrate–N. The annual N loading was determined by stream flows and concentrations on an annual basis from 1989 to 2015 and will be discussed in the “Nitrate–N loading used for calibration” section.

## Results and discussion

In this study, the observed series of the stream N dynamics will contribute to interpreting and building on the long-term trajectories of N use. The ELEMeNT model uses a limited number of parameters to simulate system behavior over extended time scales with a small amount of data. The data set out in the previous section were used to create soil surface N surplus trajectories for the years 1800–2016 across the Mondego River Basin (procedure explained in Van Meter et al. (2017)). In fact, the use of the ELEMeNT model approach allows us to quantify the long-term watershed-scale N fluxes and then to determine the N legacy and its effects on water quality.

The conceptualization of the ELEMeNT model guides it to an integrated analysis of the whole river basin. Therefore, the results are presented and commented on for that area.

### N surplus trajectory

**Fig. 7:**
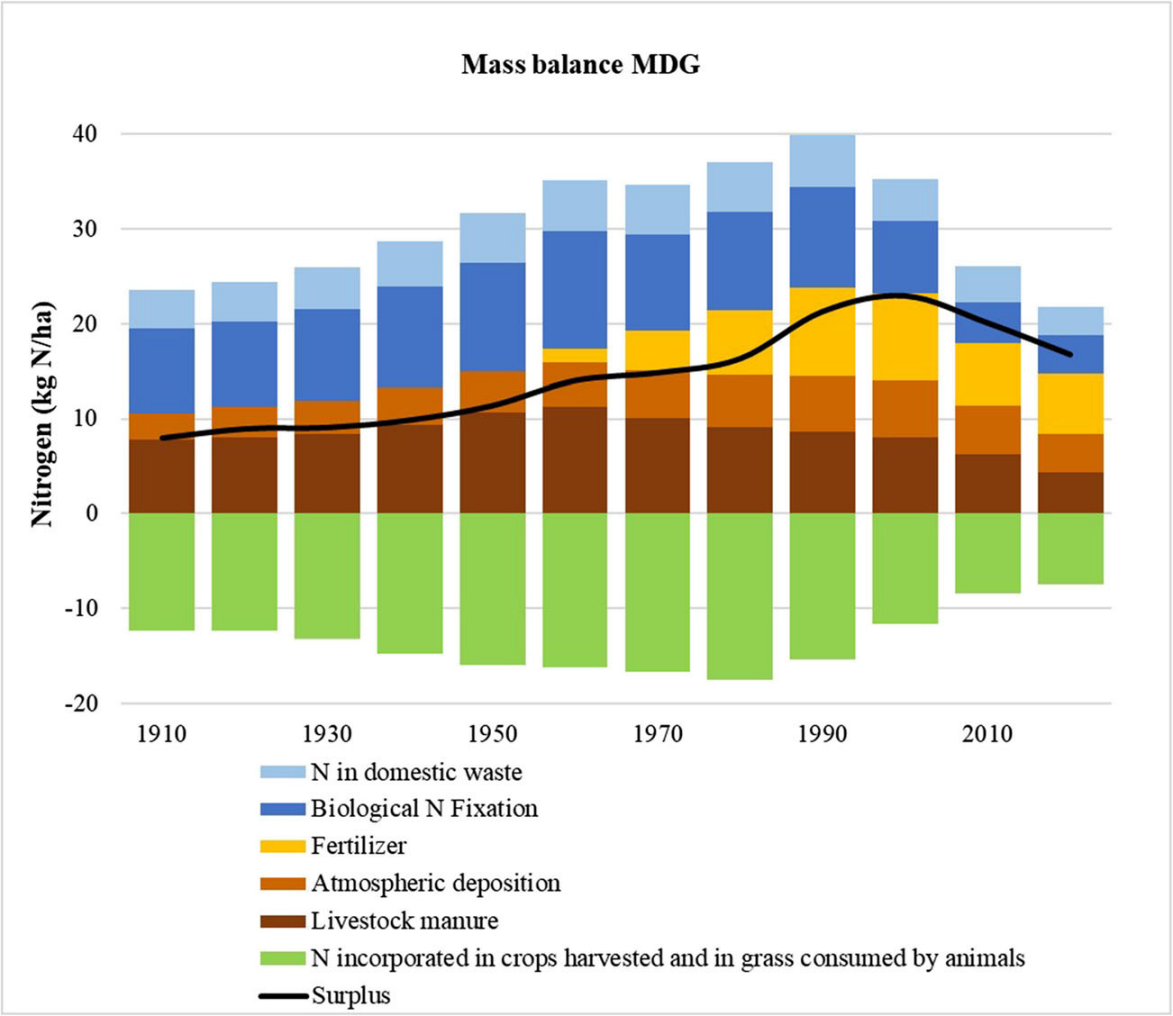
Nitrogen inputs and outputs to the MDG watershed for the period 1900–2016 and the N surplus trajectories

The surplus trajectory is represented by the dark line in Fig. 7 that can be characterized by three main trajectories over time. The first one of 50 years (from 1900 until 1950) reveals a smooth variation for the N surplus with values continuously lower than 12 kg N/ha/year. In fact, during this period, it seems that no significant changes occur in terms of N loading in the Mondego (MDG) watershed. Then, after 1950, there was an increase in the N inputs trajectory until the beginning of the nineteen-nineties. This was mainly driven by increased inorganic fertilizer application. The peak occurs in 1993 with a value of 24 kg N/ha/year for the N surplus. This value is 2 times higher than the N surplus in 1950 (12 kg N/ha/year). This peak corresponds to the same point in time as the highest level of inorganic fertilization. The last trajectory from 1993 to 2016 is characterized by a fall to N surplus values of 16 kg N/ha/year in 2016. This trajectory is driven by the reduction of most of the N input components in the MDG watershed.

In terms of the individual components for the N mass balance (Fig. 7), the manure (brown) associated with livestock rises until the end of the 1950s and then starts to drop until 2016. In the past century, the bovine and equine species were used as working animals in farming activities. As stated by Hardesty and Box (1984), cattle were used for work and to give milk and meat in small mixed farm systems. In the 1950s, after many years of economic stagnation, Portugal entered a period of robust growth and modernization until the 1970s (Lains 2001). During this period, the number of animals starts to decrease, possibly because these mixed farm systems were in decline and the animal workforce was being replaced by agricultural machinery, like tractors.

The atmospheric N deposition (orange) increases its contribution to the N surplus until the end of the second millennium to values near to 6.4 kg N/ha/year. According to Engardt et al. (2017) this was associated with the industrial revolution and the spread of private and commercial diesel fuel vehicles with high nitrogen oxide emissions. After the end of the millennium, the N deposition starts to decrease due to the implementation of measures to reduce air pollution emissions by using more environmentally efficient technologies in industry, agriculture, and transportation (Engardt et al. 2017).

Inorganic fertilization (yellow) was the main driver for an increase in the N surplus since 1950. In fact, according to Carmo et al. (2017), the use of inorganic fertilizers become essential since there was a deficit in N in agricultural land and in the 1950s, farmers started to add inorganic fertilizers to the manure already used. The use of the inorganic fertilizers increased until the beginning of the nineteen-nineties in response to the farmers’ goal of increasing the crop yield. Then there was a progressive reduction associated with BMPs based in European policies such as the Water Framework Directive and the European Community Nitrates Directive, which were adopted to limit the amounts of fertilizer use. This was linked with the innovation of farming practices that made it possible to maintain or even increase crop yields and at the same time reduce fertilizer use. The application of chemical fertilizer continued to decrease until the end of the 2000s, and since then their use has stabilized.

The biological N fixation term is mainly related to the pasture areas and the areas that are occupied in crop cultivation with biological N fixation capacity. In recent decades, these areas have reduced as can be seen by the decline of this N in the last few years, in the blue bars of Fig. 7. The leguminous plants with capacity to fix N considered in this work to determine the biological N fixation are beans, chickpeas, broad beans, and peas. The areas occupied by these crops have been losing coverage to percentages of 1% of the watershed areas in 2016. Most of these crops are cultivated to meet the family needs of subsistence farmers. But there has been a pronounced decline in subsistence farmers. According to DGHEA (1983), in the 1980s, 61% of the farmers were more than 50 years old and 16% were over 65 years old. These were not replaced by younger generations, as stated by GTAA (2003).

The last component of the N input is related to the waste production by the residents (light blue bars of Fig. 7). This component is a function of the change in the number of residents that was discussed, taking into account the expansion of the wastewater treatment plants in MDG.

The green bars represent the removal of N by crop production and by animals’ consumption of grass in pastureland. This N removal increased until the end of the 1970s when it started to decrease. The increase of crop production between 1900 and the end of the 1970s was mainly achieved by an increase in the areas under cultivation. Furthermore, the increase of inorganic fertilization and the modernization of the agricultural sector also impacted the rise in productivity and thus the N removal from crops after the 1950s. After the 1980s, crop production starts to fall, mostly influenced by the decline in agricultural activities in the MDG basin. The work of GTAA (2003) indicates reasons for this decline are related to the low return on crop cultivation, the difficulty of finding labor for agricultural work, and higher salary opportunities in other sectors.

### ELEMeNT calibration results

#### Nitrate–N loading used for calibration

**Fig. 8:**
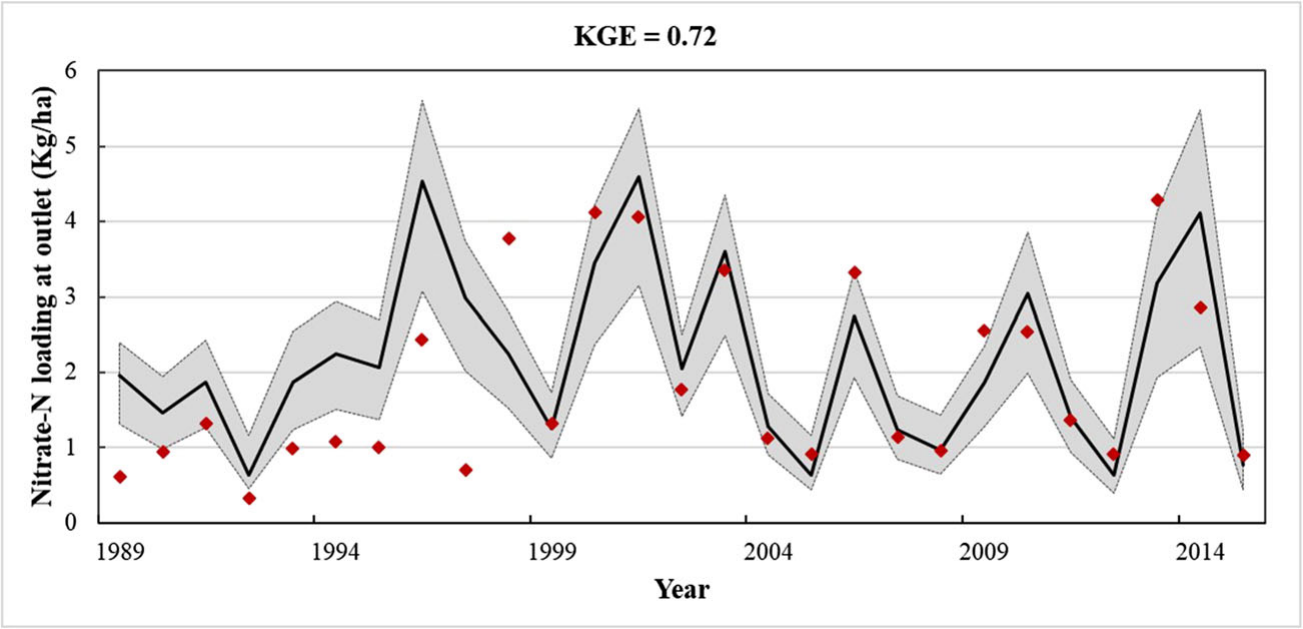
NO_3_–N loading at Mondego River Basin watershed. The red diamonds include the measured NO_3_–N values and the dark line represents the modeled values. The gray areas defined by the dash line include the 95% confidence interval given by the values of parameters after the calibration

After gathering the information to calculate the N surplus, the ELEMeNT model is calibrated by fitting the set of 10 parameters based on the comparison between the simulated and the observed N loads at the river outlet. The stream flow and concentration data were used to determine the observed annual N loads (Fig. 8). These loads are impacted by the N legacies from past agriculture practices. In this figure, the modeled results are represented by the dark line. The gray areas defined by the dashed line is the 95% confidence interval given by the parameter values after calibration. The KGE metric reaches a good value. This is a reliable metric as it provides an interesting diagnosis of the model performance taking into consideration three different types of model errors related to the mean, variability, and dynamics (Pool et al., 2018). This is important in this problem to reproduce the temporal dynamics (measured by the Pearson correlation coefficient in Eq. 5) as well as preserving the distribution of N loads (measured by variability and mean in Eq. 5). There is no unique threshold value of KGE that fits all applications (Knoben et al., 2019). Krajewski et al. (2020) considers that model provides good results if KGE > 0.5. A prior application of ELEMeNT also considers that results are reliable for a KGE > 0.5 (Liu 2020). Here, this criterion is met in a large margin. Furthermore, the sensitivity analysis (described in the “Sensitivity analysis” section), performed to deal with parameter uncertainty issues, gives additional confidence in these results.

Analysis of the figure let us to argue that the model was able to estimate the N loadings in the period under calibration (1989 to 2015).

**Fig. 9:**
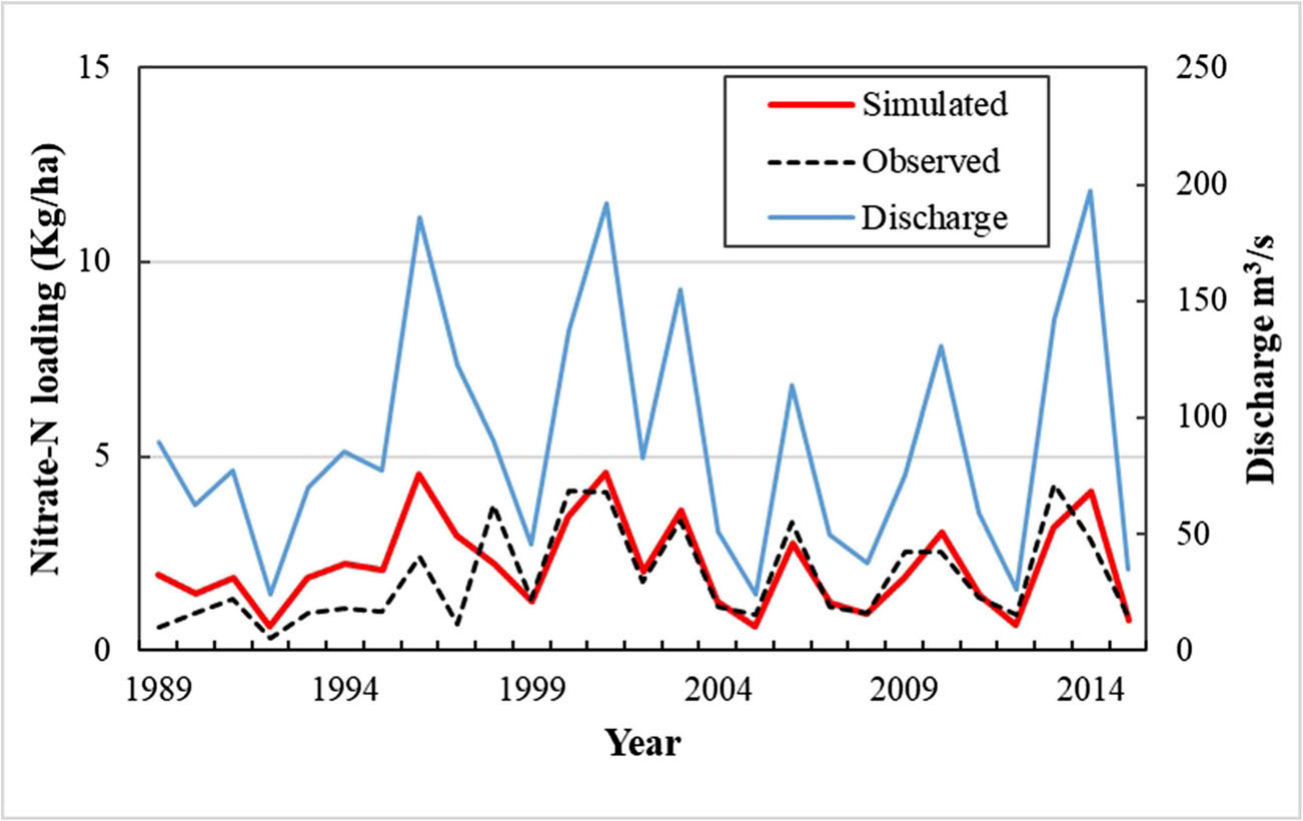
Simulated and observed nitrate–N loading and river discharge at Mondego River Basin watershed

ELEMeNT allows us to reproduce the nitrate–N loading at the outlet of the MDG watershed. Figure 9 shows the plots for the simulated and observed N loads and the river discharge for the period of years with available water quality analysis data (1989–2015). The simulated load (red) is very similar to the observed load (black) for the period from 1989 to 2015, with a modeled mean of 1.48 ktons NO_3_^−^N/year compared with the measured loadings of 1.28 ktons NO_3_^−^N/year. For the MDG, the KGE is equal to 0.72 indicating small mean, variability, and dynamics errors, which means that model successfully estimated the observed loads.

There is a deviation between modeled and measured loads in 1998, and this can be associated with wildfires that occurred in the Mondego watershed. Fires affect the concentration of nutrients in the surface waters during and after a fire. According to Ranalli (2004), these effects are most likely to occur when the fire is severe, when windy conditions accompany the fire, when there is heavy rain after the fire, if the watershed has steep slopes, and if the soils have little cation exchange capacity. In the Mondego, in that year, most of these conditions could have converged and impacted the concentration of nutrients in the surface waters.

#### Model parameter description

The calibration results can be found in Table 1 and include, for each parameter, the calibrated median values obtained by OSTRICH and the minimum and maximum ranges. The nitrogen content in the soil in pristine conditions (*M*_s_), soil porosity (*n*), and soil water content (*s*) are parameters related to the physical characteristics of the MDG watershed and values obtained are in line with those available on the LUCAS soil database (Orgiazzi et al. 2018). The amount of N accumulated as biogeochemical legacies is a function of the organic N mineralization rate, which is conceptualized in ELEMeNT as a first-order process with a rate constant of the active pool (*k*_a)_. This rate is equal to 0.14 per year for the Mondego basin, which is similar to 0.11 per year for the Mississippi River Basin (MRB), and 0.13 per year for the Susquehanna River Basin (SRB) (Van Meter et al. 2017). The soil denitrification rate constant (*λ*_s_) equal to 0.58 per year in the Mondego, which is higher than the value determined for MRB (0.54) and SRB (0.57). This is reasonable given the MDG is in the Mediterranean region and higher temperatures lead to increased denitrification rates. In addition, in the lower Mondego, there are areas dedicated to the rice cultivation, and it is recognized that rice paddies are important denitrification spots. Regarding the protection coefficient of cultivated land (*h*_c_), this is equal to 0.17 for MDG and the protection coefficient of non-cultivated land (*h*_nc_) is equal to 0.43. These values are lower than MRB (0.37) and SRB (0.41) for the *h*_c_ coefficient and lower than MRB (0.48) and SRB (0.6) for *h*_nc_. These coefficients are used to determine the part of the N inputs that enter the active pool and those that enter the protected pool, and the higher the value, the greater the percentage of the N inputs that enter in the protected pool. The low values for these coefficients in the MDG basin lead to more N inputs in the active pool with faster reactions than in the protected pool. This reduces biogeochemical legacies compared with MRB and SRB. The mean groundwater travel time (*μ*) is a very important parameter that captures the hydrological time lags. This value is equal to 25.5 years for the Mondego, higher than MRB’s 16 years and SRB’s 15.6 years. It is related to the physical properties of the MDG aquifer in terms of the gradient driving flows that are different from those of the MRB and SRB. The groundwater denitrification rate constant (*γ*) with a value equal to 0.05 per year is much less than the 0.11 per year of the MRB and 0.27 per year of SRB. This parameter describes the removal of N via denitrification in the groundwater paths. In the MDG, the denitrification amount via groundwater is not as relevant as it is for the MRB and SRB. Finally, the value of the human denitrification rate constant (*k*_pop_) is equal to 1 per year in the Mondego, higher than the value of 0.83 per year for MRB and SRB. This means that in the Mondego basin, it is hypothesized that the entire contribution of the human population is removed by denitrification.

**Table 1:** Calibrated parameters and range minimum and maximum values

Parameter	Calibrated median	Range—min	Range—max
*M*_s_ (kg/ha)	3990	3740	5920
*k*_a_ (year^−1^)	0.14	0.09	0.17
*n*	0.51	0.41	0.53
*s*	0.66	0.29	0.73
*λ*_s_ (year^−1^)	0.58	0.27	0.75
*h*_c_	0.17	0.14	0.26
*h*_nc_	0.43	0.28	0.52
*μ* (year)	25.5	10.4	27
*γ* (year^−1^)	0.05	0.01	0.19
*k*_pop_ (year^−1^)	1.00	0.87	1.00

### Nitrogen stores and fluxes

**Fig. 10:**
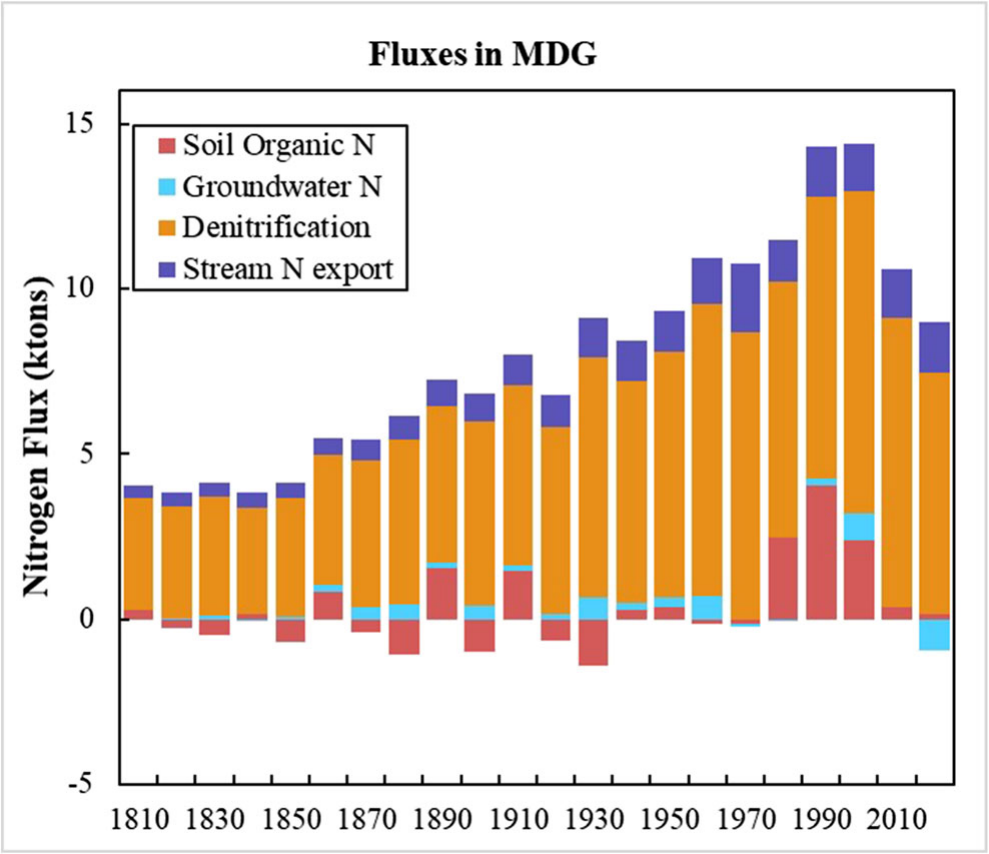
Variation in nitrogen fluxes in the MDG watershed between 1800 and 2016

Of the N surplus accumulated in the MDG basin, one part leaves the watershed by the riverine N fluxes and by the denitrification processes in soil and groundwater and in the wastewater treatment plants; the other part remains in the soil and groundwater stores. The N fluxes in the MDG watershed quantified by the ELEMeNT model are shown in Fig. 10.

The highest N losses across all periods are from denitrification. These are higher than the losses from the stream N export in the river outlet. A high proportion of N loss due to nitrification is common in watersheds with high forest cover areas, as in the MDG case (Fang et al. 2015). Furthermore, according to Romero et al. (2016) warm temperatures increase the denitrification rates and the MGD can be described as a warm watershed. They also note the effect of large reservoirs like the Aguieira dam in the upper MDG, which can also favor the increase of denitrification processes. In addition, Van Meter et al. (2017) show a high proportion of denitrification contribution for the Susquehanna River Basin. This is a basin with large forest areas and percentages of cropland areas like those of the MDG. For the Mississippi River Basin, also analyzed, the situation was different, since this is a basin where there is intensive land use by croplands and pastures.

Between 1800 and 1966, some soil organic N fluxes are found on the depletion side (lower than zero). This is associated with the soil as a source of mineralized N for surface crops and groundwater. The same kind of depletion soil N fluxes were observed in Van Meter et al. (2017). After this period, the soil become an N sink, since the N surplus increased in line with the increased fertilization of cropland. This could also be related to less land being used for agriculture in the MDG basin. Regarding the groundwater fluxes, Fig. 10 includes a net positive N flux for most of the entire 1800–2016 period, that is associated with soil as a source of mineralized N and with the N surplus increase.

### Nitrogen stores in soil and groundwater

**Fig. 11:**
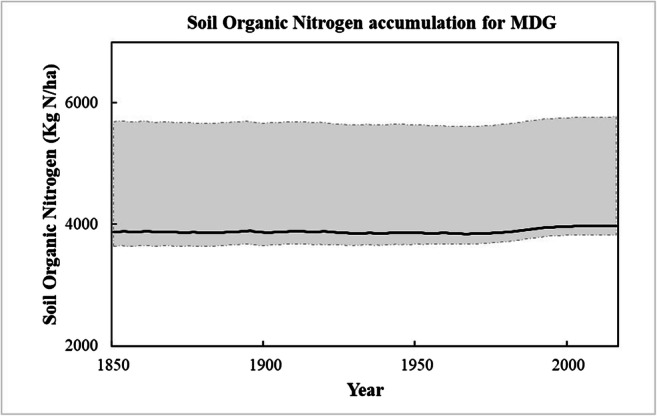
Soil Organic Nitrogen accumulation between 1800 and 2016 in the MGD watershed

The N surplus that is generated in the MDG watershed from 1850 to 2016 is 1460 ktons of N. Of this input, 78% left the MDG watershed by denitrification and more 13% by the riverine outputs. Only 9% of this N input remained in the watershed in soil and in the groundwater store. In terms of soil, the mean values of analysis from the same 21 MDG points in 2009 (LUCAS soil database, Tóth et al. 2013) and in 2015 (LUCAS soil database, Orgiazzi et al. 2018) are very similar. From these databases, a mean value of 1.69 g N/kg of soil is determined in 2009 and the similar mean value of 1.68 g N/kg of soil in 2015. Figure 11 shows the small variation in Soil Organic Nitrogen accumulation between 1850 and 2016 estimated by ELEMeNT.

**Fig. 12:**
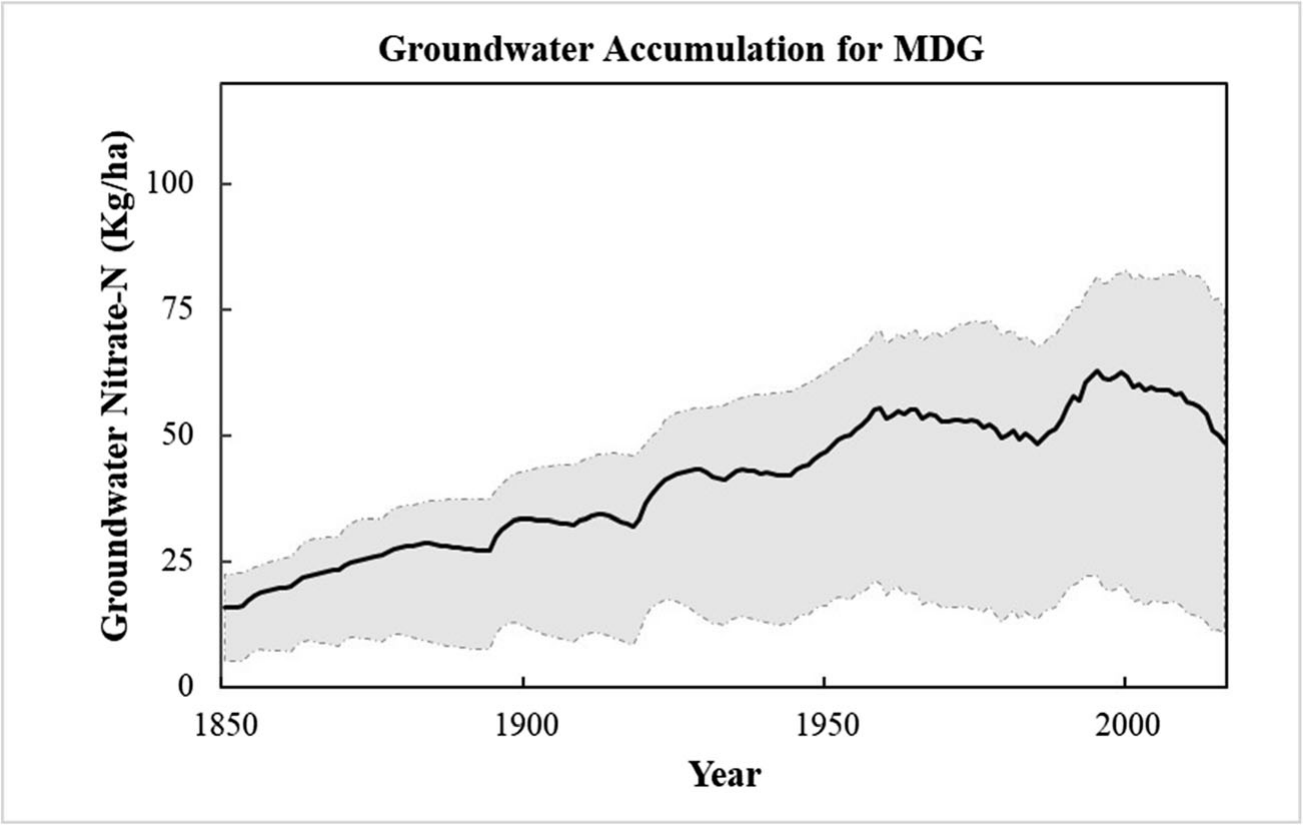
Groundwater N accumulation between 1850 and 2016 in the MGD watershed

The groundwater in the MDG basin shows a constant N accumulation (Fig. 12) from 1850 to 2016. This is consistent with the increase in groundwater concentration in the NO3 N analysis consulted at SNIAmb (2020), which included values of 4 mg NO /L in 2003 and growth to 5 mg NO /L in 2015, measured at a station in the MDG area.

The same kind of net positive groundwater N accumulation was reached in other studies. In the work of Van Meter et al. (2017) the Susquehanna and Mississippi River Basin also show a positive N accumulation in groundwater and the authors found that there is a consistent relationship between the N accumulation rate and the level of groundwater recharge.

### Legacies in the MDG watershed

The legacies were conceptualized by ELEMeNT as hydrological and biogeochemical. As stated, the hydrological legacies are associated with the groundwater travel time distributions across that watershed and the biogeochemical legacies are a function of the N accumulated in the soil and the organic N mineralization rates.

**Fig. 13:**
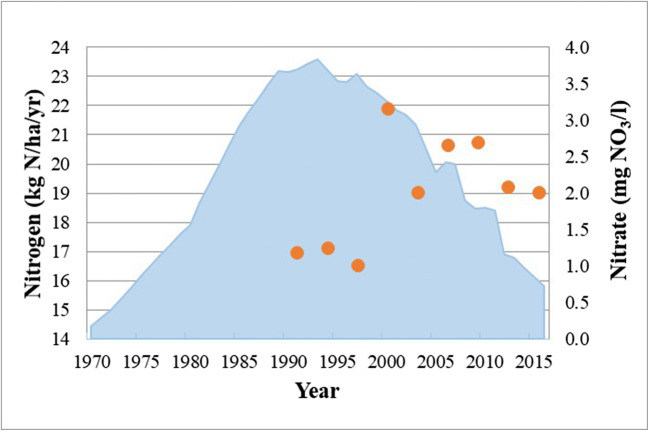
Trajectories of the N surplus between 1970 and 2015 (light blue) and nitrate values from 1989 to 2015 (orange points) determined as weighted values for intervals of 3 years

Accumulation of these legacies have indeed contributed to time lags in water quality improvement. This is apparent if we look at Fig. 13. Based on this figure, the N surplus values for the MGD basin (light blue area) increased from the 1970s but then started to decrease after the beginning of the 1990s until 2015.

The N surplus has decreased due to several reasons, which were explained in detail in the “N surplus trajectory” section as the reduction of farming areas and the implementation of BMPs in the agricultural sector. The best management practices were mainly related to legislative constraints on the application of inorganic and organic fertilizers. If fact, the inorganic fertilization obtained for the Mondego region from the EC (European Commission), at the Joint Research Centre of the European Union directory (https://water.jrc.ec.europa.eu/), documented a decrease since the early 1990s. This was related to the legal constraints for the protection of water bodies, as a result of innovations in the agricultural sector, such as precision agriculture, and also related to economic constraints as suggested by Cameira et al. (2019). Livestock density also decreased in the MDG watershed due to the abandonment that has occurred in the agricultural sector in recent decades (Cunha et al. 2021). Despite the decrease in N inputs, the nitrate concentrations at the watershed outlet (orange points) had a monotonically increased trend till first decade of the new millennium, and after which, it started decreasing. These points were obtained weighing nitrate concentrations with discharge flows over a time period based on the data introduced in the “N loads in the stream” section and are represented in Fig. 9. As such, the effect of interannual discharge flow variation in long-term analysis of nitrate concentrations is reduced (Van Meter and Basu, 2017). These differences arise due to nutrient legacy effects in the MDG watershed. Indeed, the peak in N concentration lags the peak in N surplus by approximately 23 years, and this is of the same order of magnitude as the calibrated mean travel time of the MDG = 25.5 years. This means that current observed N loads at outlet are also impacted by the N applications at two decades ago. The soil and groundwater legacies accumulated in the watershed would probably maintain these concentrations over longer periods.

## Conclusions

The capabilities of the ELEMeNT model were presented and fully explored to understand the effects related to the N anthropogenic loads and to quantify the corresponding N legacy at watershed level. The ELEMeNT model was able to capture the changes in land uses and the evolution of the agriculture practices and determine the nitrate load trajectory at the outlet with a high KGE of 0.72. Given that the Mondego is not an agriculture-dominated watershed, unlike the MRB, SRB, and Grand River basin that were studied by ELEMeNT (Van Meter et al., 2017), it is encouraging that the model was able to capture the dynamics over this watershed.

Exploration of the N inputs and outputs using the ELEMeNT model allows us to gain critical insight about watershed processes. The N surplus drawn trajectory shows no major change from 1800 until 1950 and then there was an increase in surplus N until the beginning of the 1990s. This was mainly driven by the increased use of inorganic fertilizer. In recent decades, the N surplus has declined. This is mainly associated to the use of best management practices mainly related to legislative constraints on the application of inorganic and organic fertilizers and with a substantial reduction of areas under agriculture. The reasons for this reduction are related to the low return on crop cultivation, the difficulty of finding labor for agricultural work, and higher salary opportunities in other sectors. A plan for the improvement of agricultural land conditions was designed but only roughly half of the planned area has been improved, and this contributed to the abandonment of some agricultural areas with very fertile soils.

A time lag of about two decades between the peaks of N surplus and the peak of N load at the watershed outlet is indicative of nitrogen legacies accumulated in the watershed. Indeed, we found that despite lack of highly intensive agriculture in the basin, soil and groundwater N legacies have accumulated and are of substantial magnitude (47 kg N/ha per watershed area in the last 30 years), compared to mean fertilization rates of 6.5 kg/ha/year per watershed area in the same period. The application of the ELEMeNT model also allowed us to conclude that the N losses from denitrification are high in the Mondego. This is reasonable given that MDG has high forest cover areas, warm temperatures and large reservoirs, factors which favor the increase of denitrification processes. The findings are relevant to other watersheds around the Mediterranean region, where similar conditions can be found.

The results obtained with the ELEMeNT model are also valuable for supporting decision making in watershed management. Currently, nitrate concentrations in the MDG are lower than the WHO standards, and nitrate is considered an important pollutant. However, as agricultural activities intensify, the situation is likely to change. Given the potential of N legacy accumulation in the soil and groundwater, it is important to consider the development of these legacies and minimize their accumulation as agricultural activities intensify. This can be done with better agricultural practices that maintain fertilization at relatively lower levels.

## Supplementary Information

ESM 1 (DOCX 395 kb)
