# Supplementary material for: Nitrogen legacies in anthropogenic landscapes: a case study in the Mondego Basin in Portugal
Source: Environ Sci Pollut Res Int. 2021 Nov 24;29(16):23919–35. doi: 10.1007/s11356-021-16725-x (PMC8979911; doi:10.1007/s11356-021-16725-x)
Supplement: Supplementary file 1 — (DOCX 395 kb) [file 11356_2021_16725_MOESM1_ESM.docx]

Supplementary Materials for:

**Nitrogen legacies in anthropogenic landscapes: A case study in the Mondego Basin in Portugal**

J. Marques^1^, J. Liu^2^, M. Cunha^1^, K. J. Van Meter^3^, N. B. Basu^2,4^

^1^ University of Coimbra, INESC Coimbra, Department of Civil Engineering, University of Coimbra, Polo 2, 3030-788 Coimbra, Portugal.

^2^Department of Earth and Environmental Sciences, University of Waterloo, Waterloo, Ontario N2L 3G1, Canada.

^3^Department of Earth and Environmental Sciences, University of Illinois at Chicago, Chicago, Illinois 60607, United States.

^4^Department of Civil and Environmental Engineering, University of Waterloo, Waterloo, Ontario N2L 3G1, Canada.

Corresponding author: J. Marques, [jmarques@dec.uc.pt](mailto:jmarques@dec.uc.pt)

**Contents of Supplementary Materials**

Introduction ………………………………………………………………………………………1

Text S1..…………………………………………………………………..……………………....1

Text S2..…………………………………………………………………..……………………....2

Text S3..…………………………………………………………………..……………………....2

References ………………………………………………………………………………………..2

Figure S1: Number of animals (thousands) in the low and upper Mondego ………………………3

Figure S2: Number of residents (thousands) in low and upper Mondego ……………………….4

Figure S3: Atmospheric N deposition in the Mondego basin …………………………..………..4

Table S1: Areas in (ha) of the different crops in the sub-basins of upper and lower Mondego (periods 1989 to 2016)……………………………………………………………………………5

Table S2: Amount of fertilizer use per crop area and per year obtained directly from the EC (European Commission), at the Joint Research Centre of the European Union directory (https://water.jrc.ec.europa.eu/)..…………………………………………………………………5

**Introduction**

This document includes supporting information with the data to assess the inputs of organic and inorganic fertilizers, human waste, atmospheric deposition, biological nitrogen fixation and the outputs of crop harvesting and pastures from grazing animals.

**Text S1**

Organic fertilization is a function of the number of animals of different species. From the INE (Statistics Portugal) (INE 2020), the livestock numbers are available for cattle, pigs, goats, sheep, equine species, poultry, and rabbits and for the years 1934, 1940, 1955 1972, 1979, 1989, 1999, 2009 and 2016.The years 1934, 1940 and 1972 correspond to general listing of livestock, the years 1955 and 1972 to surveys of farming activities, the years 1979, 1989, 1999, and 2009 correspond to National Agricultural Census (INE-NAC) and the last year, 2016, from INE Agricultural Statistics (INE-AS) available in INE (2020). In fact, at the present date, the 2019 agricultural census is still to be published. The animal species considered and the variation of the number of animals (in thousands) in low and upper part of the Mondego river are included in Fig. S1. Cattle increased from 1934 until 1979 in low Mondego and until 1989 in upper Mondego, and then started to decrease until 2016. For pigs, the number of animals increased from 1934 to a maximum in 1972 in upper Mondego and to 1989 in low Mondego. The number of pigs was relatively stable between 1972 and 1999 in low Mondego and then decreased until 2016. In terms of goats and sheep, the number was high in 1934, 1940 and 1955 and then declined in both the low and upper parts. The reduction was as much as 66% from 1934 to 2016. The number of horses follows the same trajectory as goats and sheep, but the reduction has been much more drastic as the number of animals of this species has fallen 91% since 1934 (Cunha et al. (2021) explains the reasons for this tendency). Regarding poultry and rabbits, there were no INE data available for 1972 and 1979. In the years with data, for both species there was an increase of livestock until 1955 and then the numbers decrease with different trajectories. In terms of poultry, large poultry farms were built in low and upper Mondego that raised many thousands of chickens. These animals were not considered because the waste from these industrial units is removed and transported to other regions of the country. Only the poultry that contribute to N inputs are considered.

**Text S2**

The crop-producing areas with the biological fixation capacity have been decreasing since 1989 based on the INE-NAC and INE-AS (INE, 2020) databases. These crops are beans, chickpeas, broad beans, and peas. The biological N fixation coefficient of each crop is based on CBPA (2018). In 1989, these crops with an area of 8400 ha (6348 ha in the upper Mondego and 2052 ha in low Mondego) represent 5% of the total agricultural areas and in 2016 this fell to just 1% with an area of 800 ha (512 ha in the upper Mondego and 288 ha in low Mondego) and showed that these crops are not commonly grown in the Mondego basin.

**Text S3**

The most important cultures in the low Mondego are maize and rice, and vineyards and olive trees in areas farther from the main river course. Potatoes were also a relevant crop in 1989 (with 4% of the total agricultural area in 1989) but they have been losing importance and stood at 1% in 2016. Together, all forage crops (oats, maize, sorghum and other forages) represent an important area across the period covered in Fig. 8. For the upper Mondego, the most important crops are maize, olives and vineyards In terms of potatoes, there was a reduction in the upper Mondego just as there was in the low part. Meanwhile, the fruit tree orchards have increased in importance, and there are larger areas of almond and chestnut trees.

**References**

CBPA - Código de Boas Práticas Agrícolas (2018). Despacho nº1230/2018 de 5 de fevereiro de 2018 do Ministérios do Ambiente e Agricultura, Florestas e Desenvolvimento Rural. Diário da República, 2ª série, nº 25.

Cunha M, Marques J, Azevedo J, Castilho A (2021). Understanding the impact of a major hydro-agricultural project in Low Mondego area (Portugal). Land 10:114. <https://doi.org/10.3390/land10020114>

INE, Instituto Nacional de Estatistica (2020), https://www.ine.pt, Accessed May 2020.

**Figure S1**


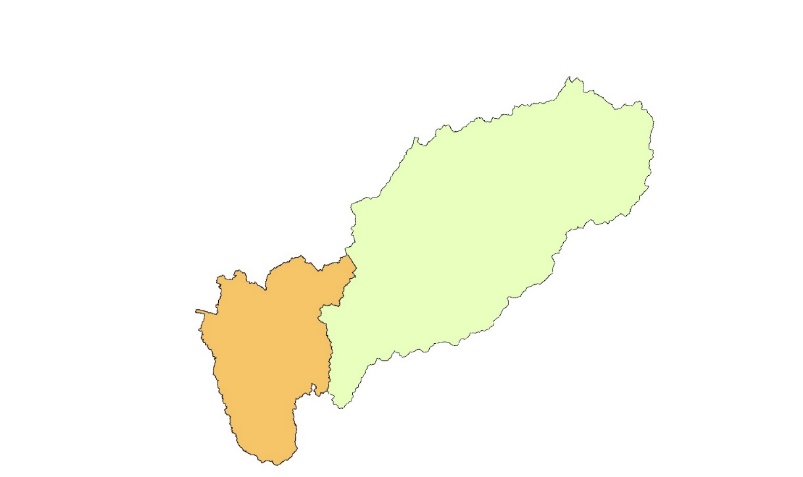

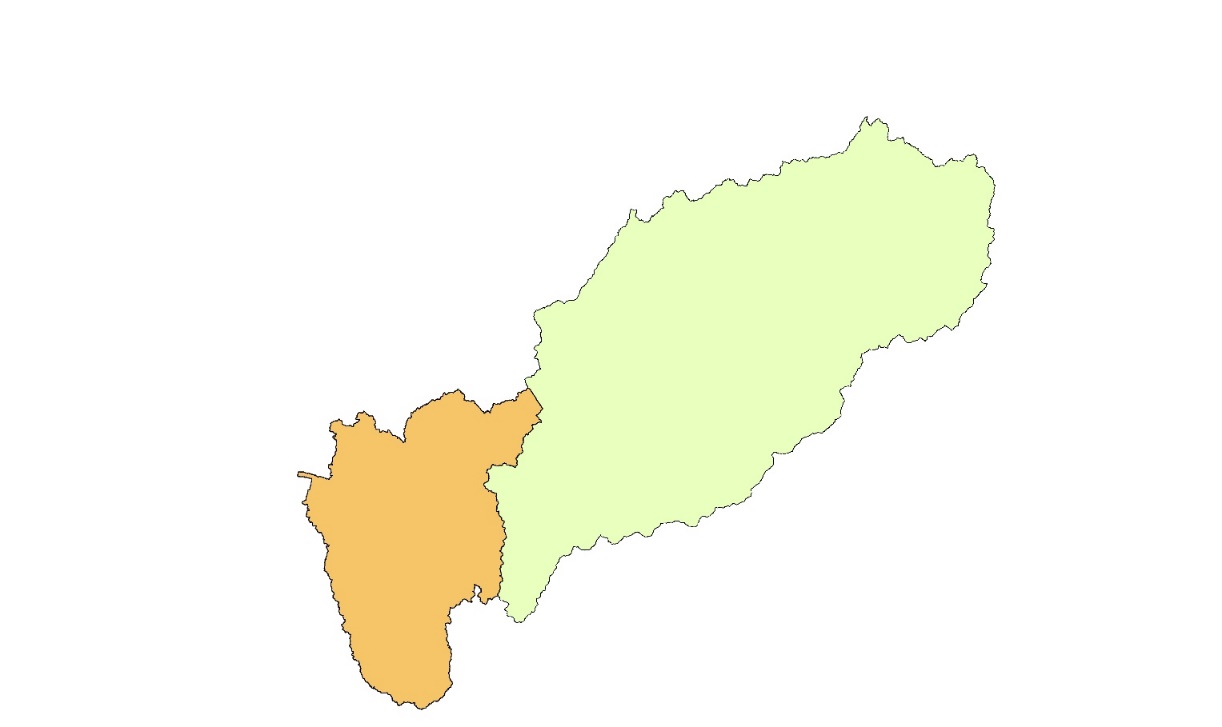

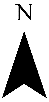


Pigs

Cattle


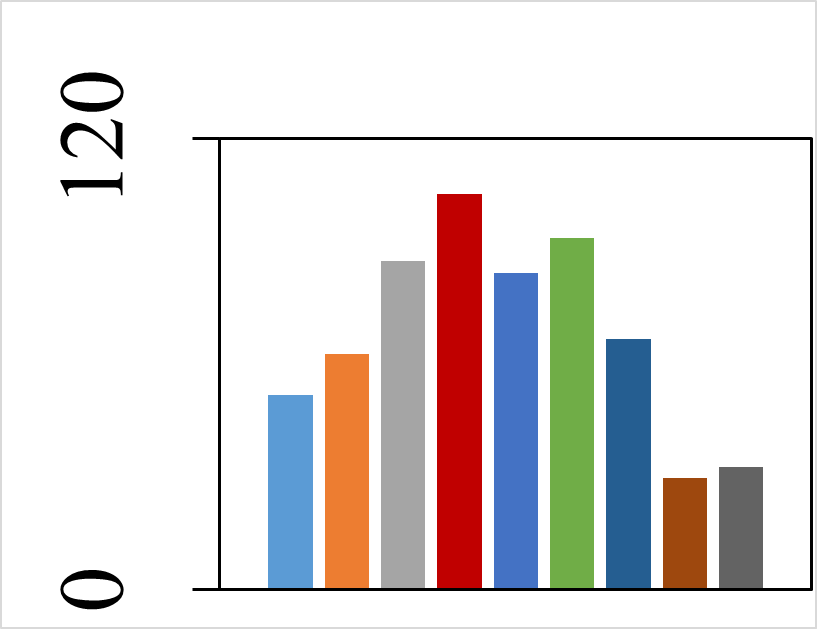

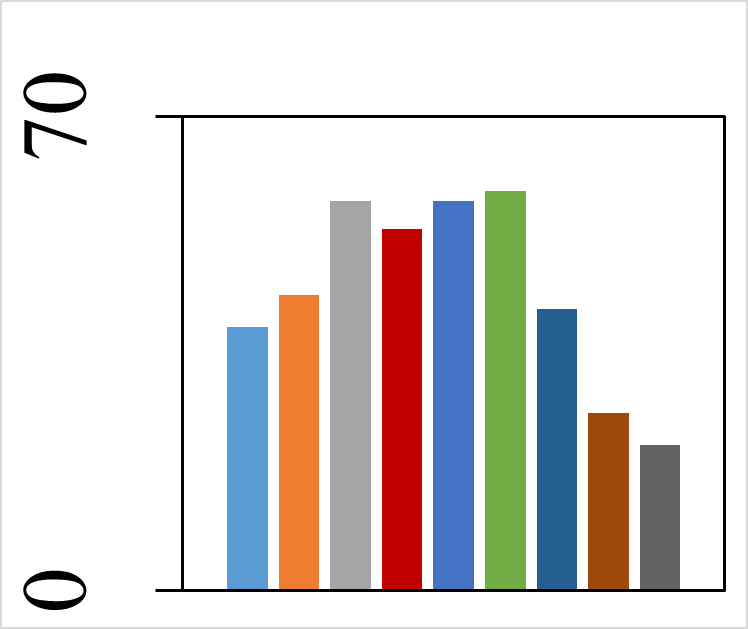


Nº Animals (x10^3^)

Nº Animals (x10^3^)


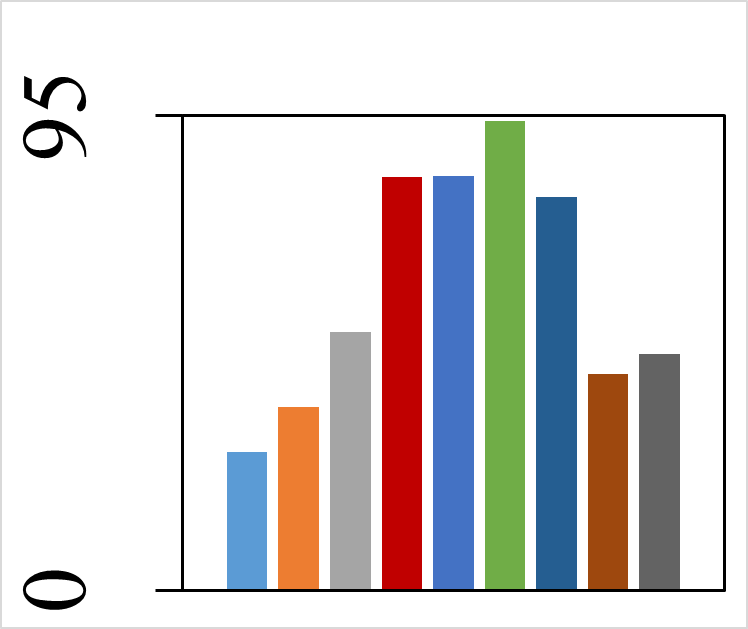

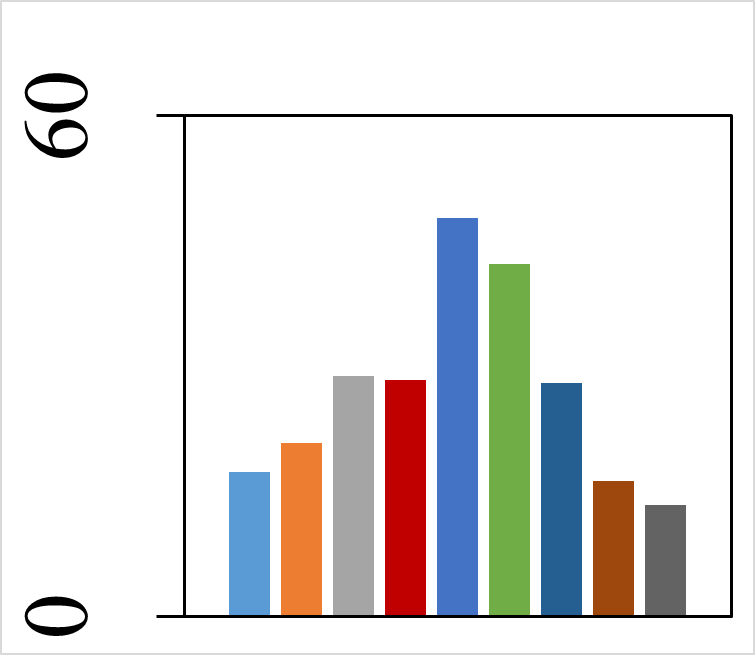


Nº Animals (x10^3^)

Nº Animals (x10^3^)

**
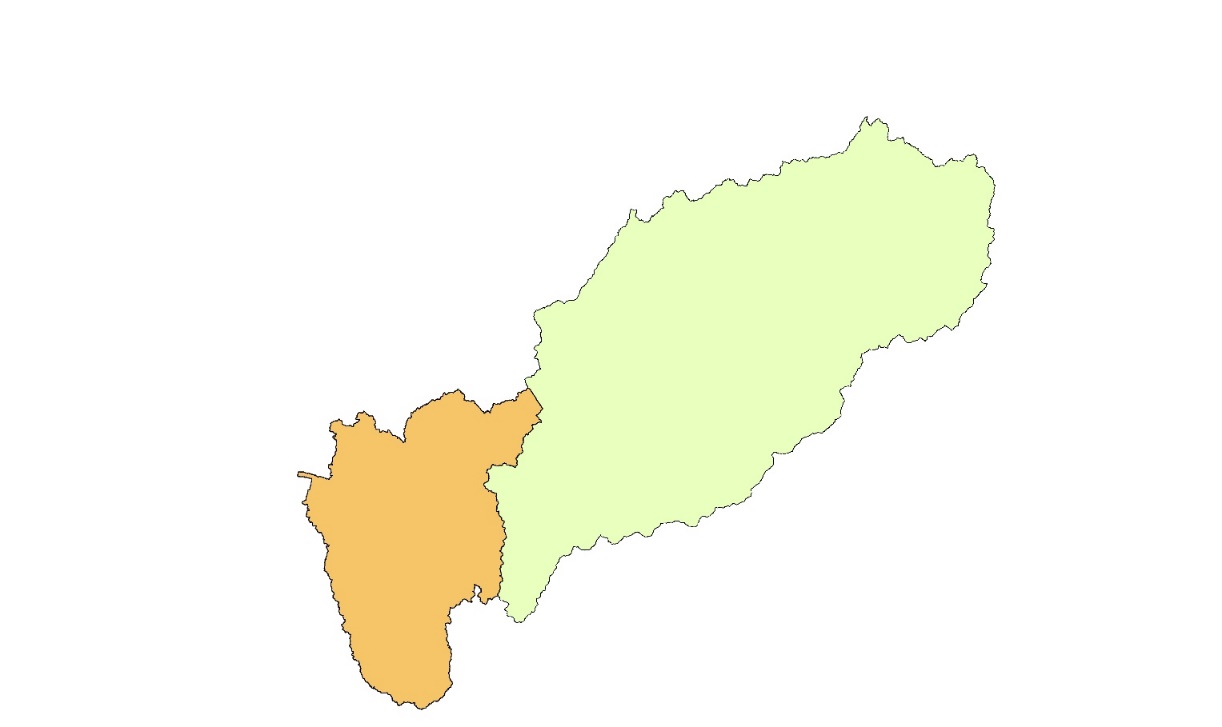

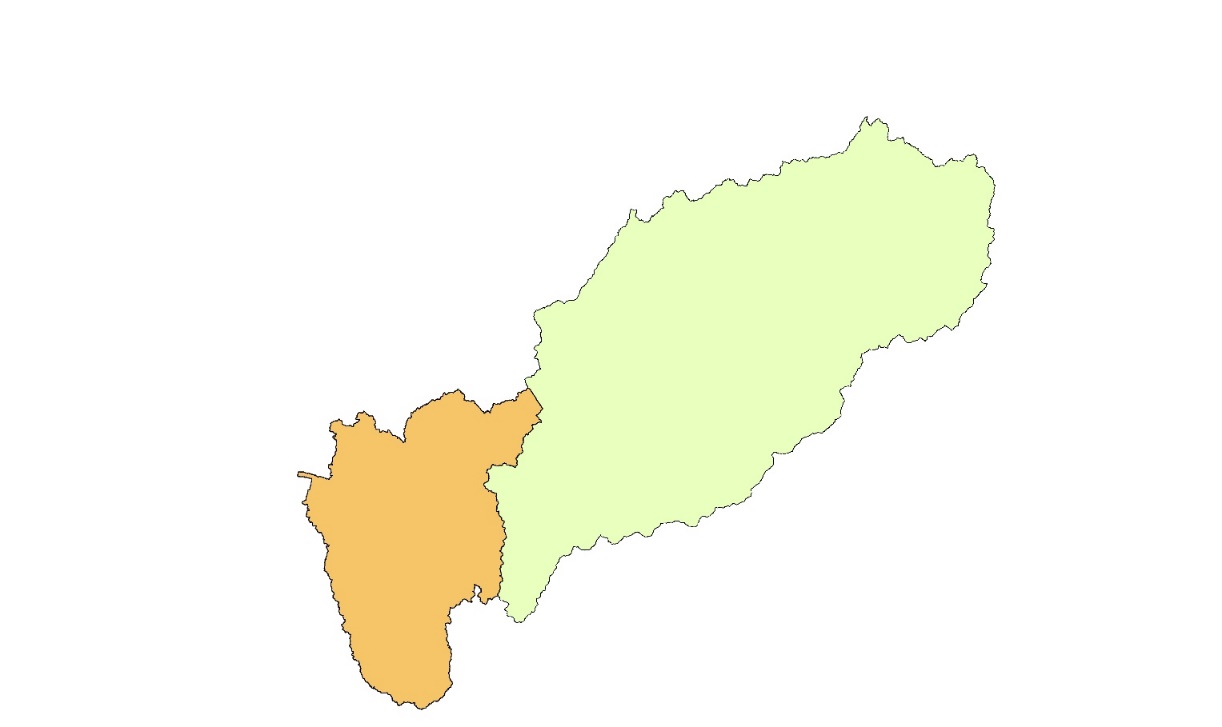
**

Equine

Goats and Sheep


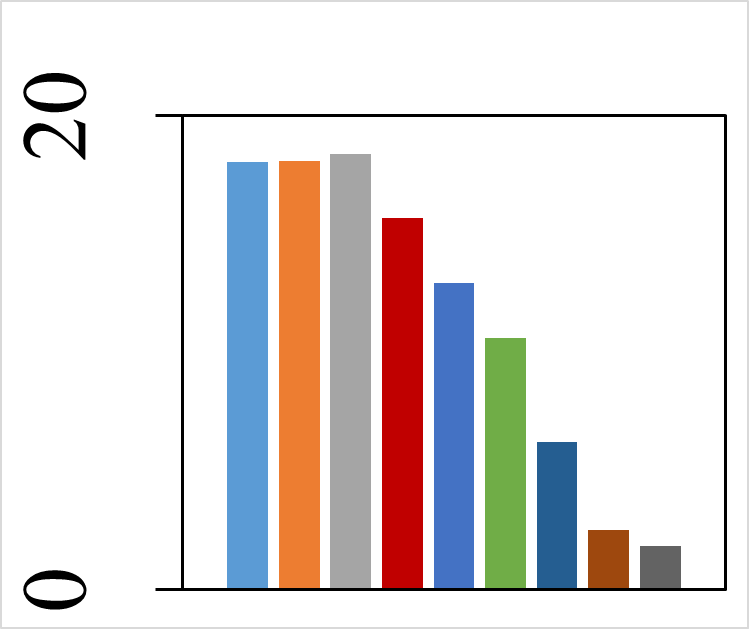

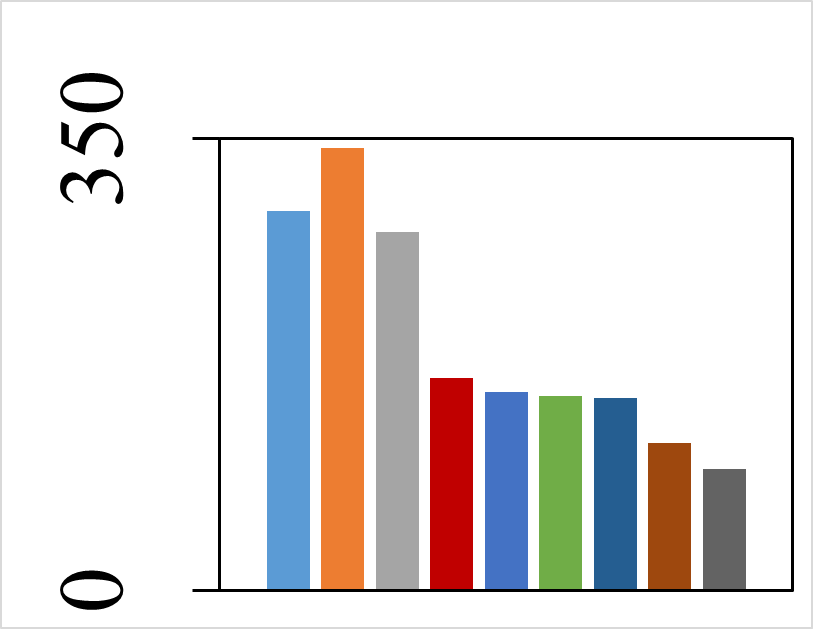


Nº Animals (x10^3^)

Nº Animals (x10^3^)


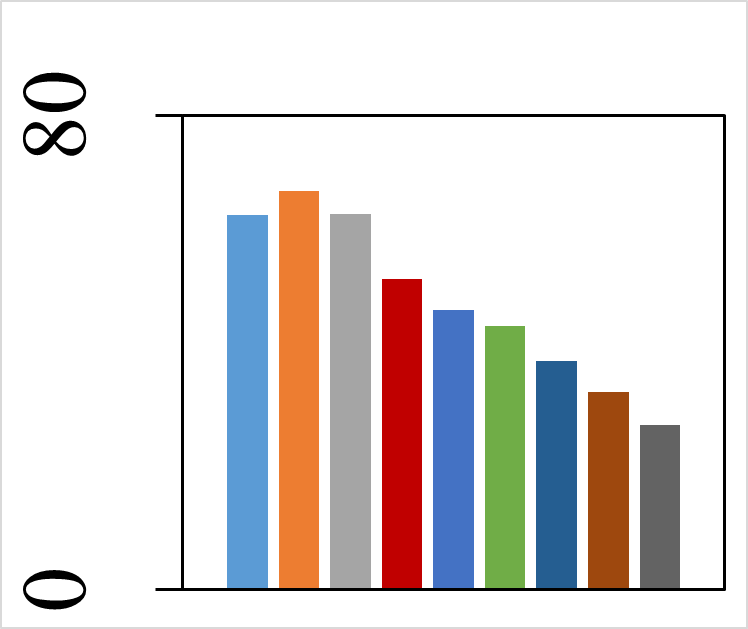

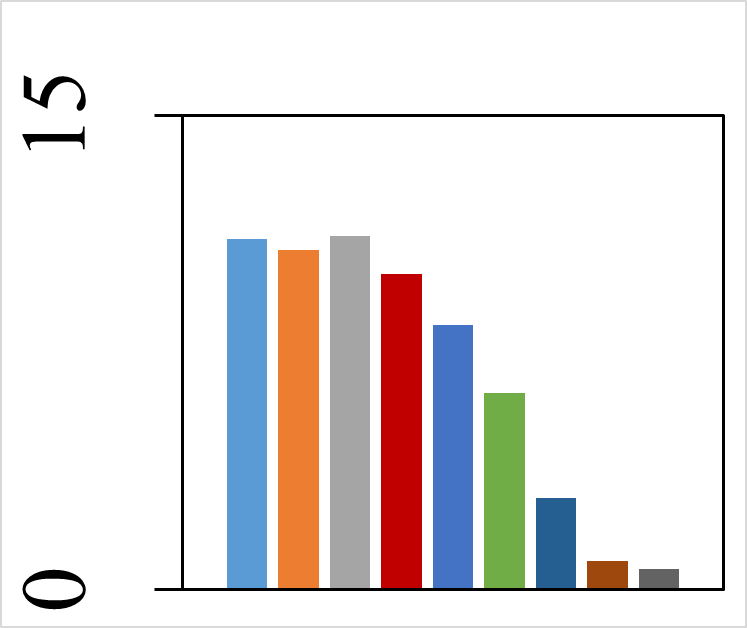


Nº Animals (x10^3^)

Nº Animals (x10^3^)

Rabbits

Poultry


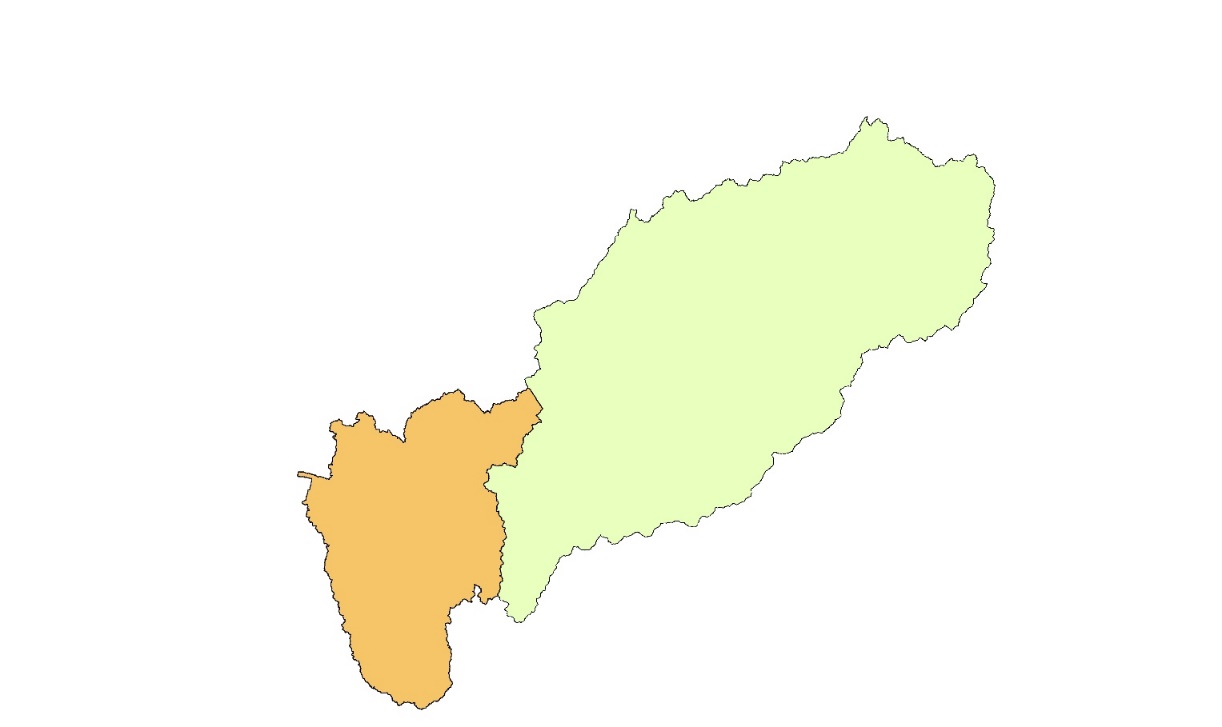

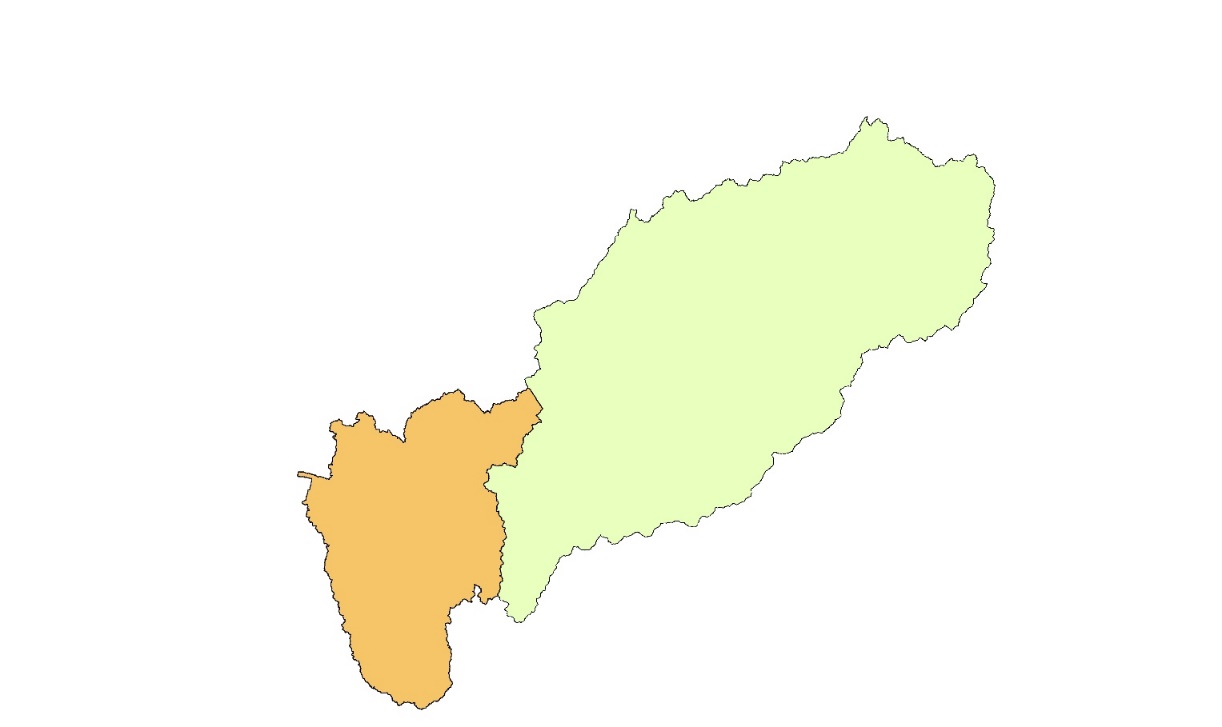


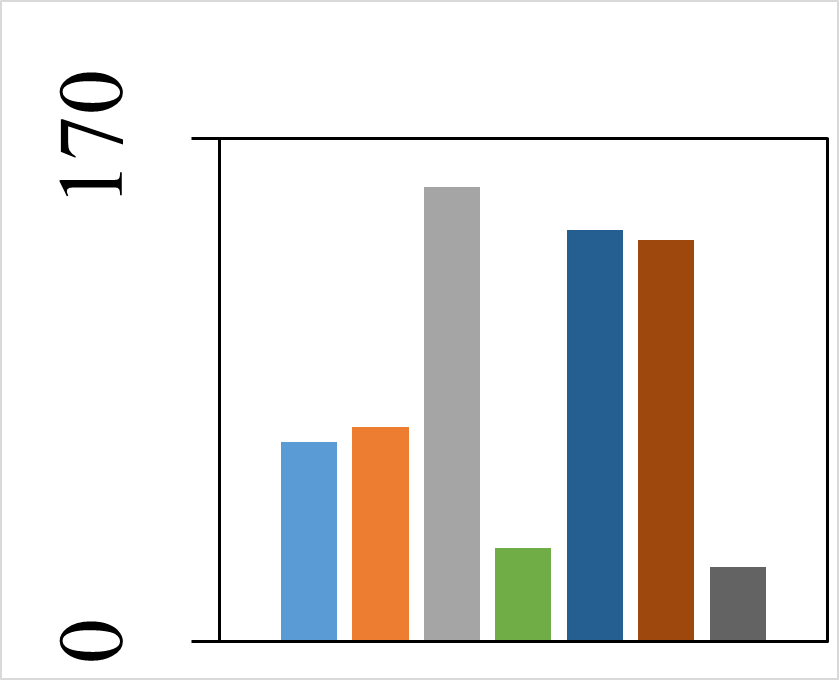

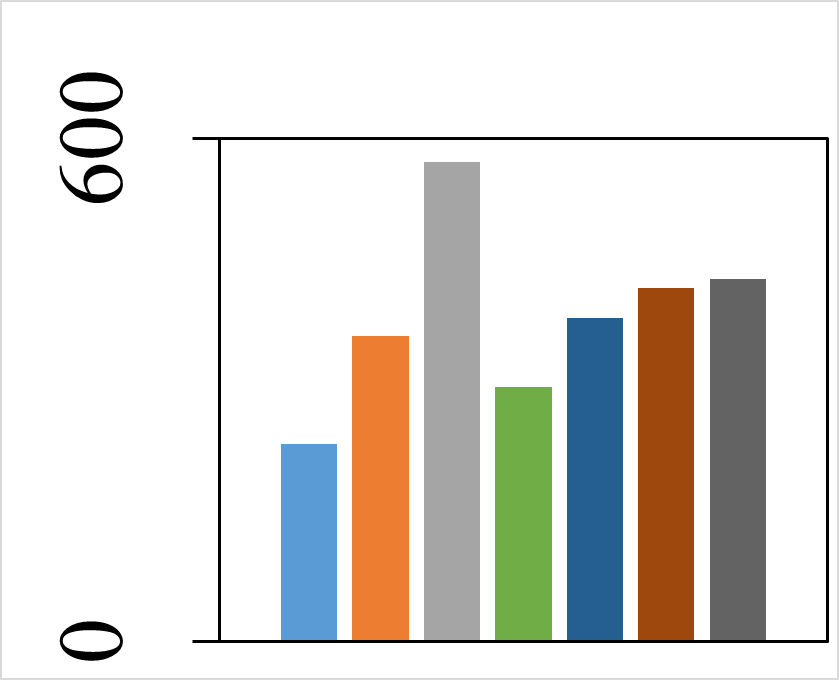


Nº Animals (x10^3^)

Nº Animals (x10^3^)


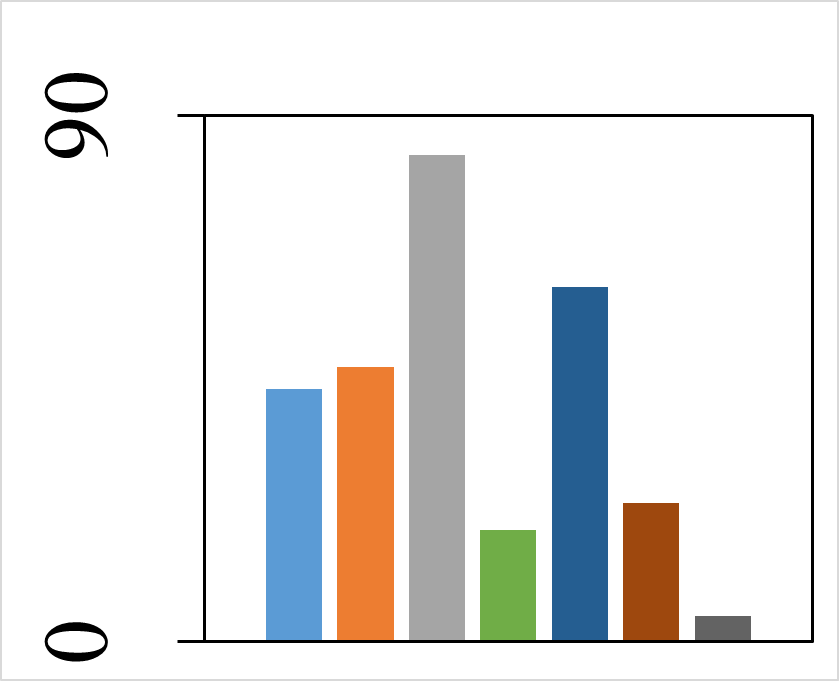

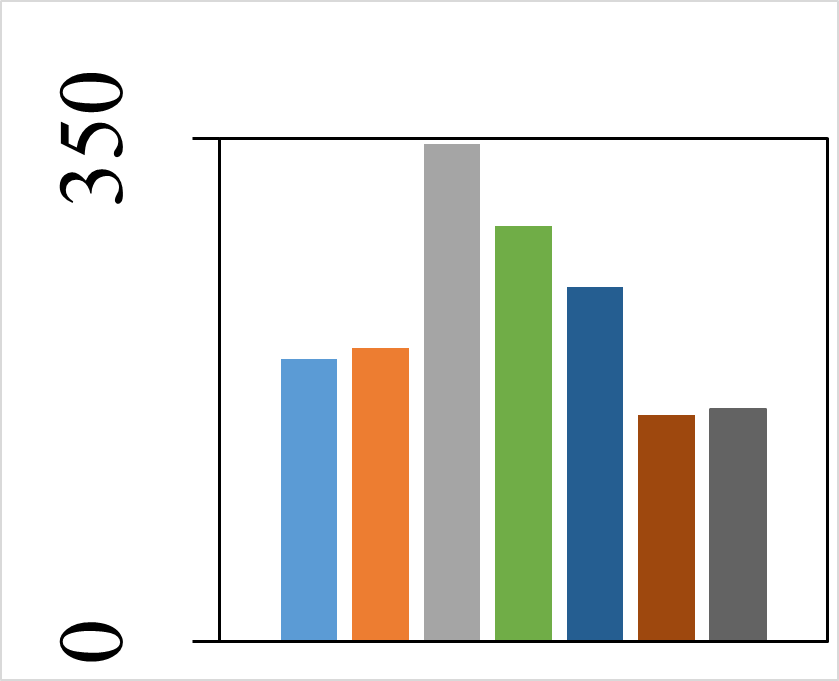


Nº Animals (x10^3^)

Nº Animals (x10^3^)

1979

1989

1999

2009

2016

1940

1934

1972

1955

**Fig. S1** Number of animals (thousands) in the low and upper Mondego

**Figure S2**


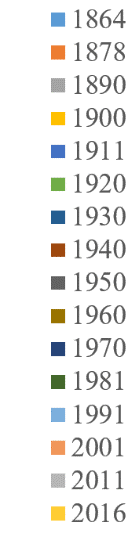

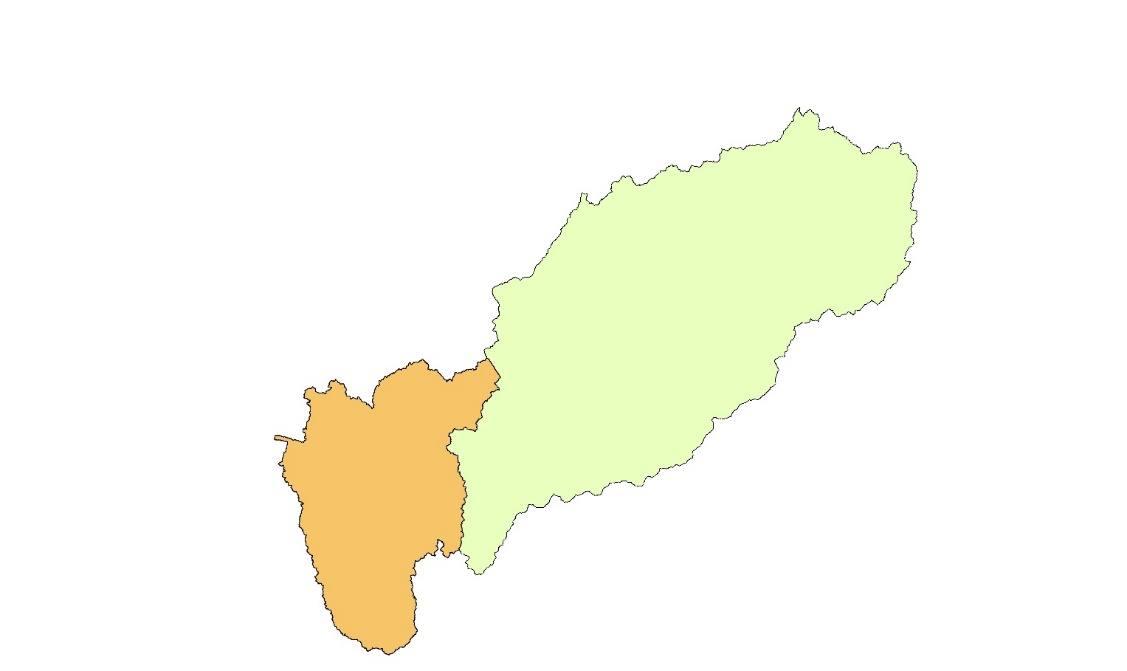

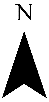


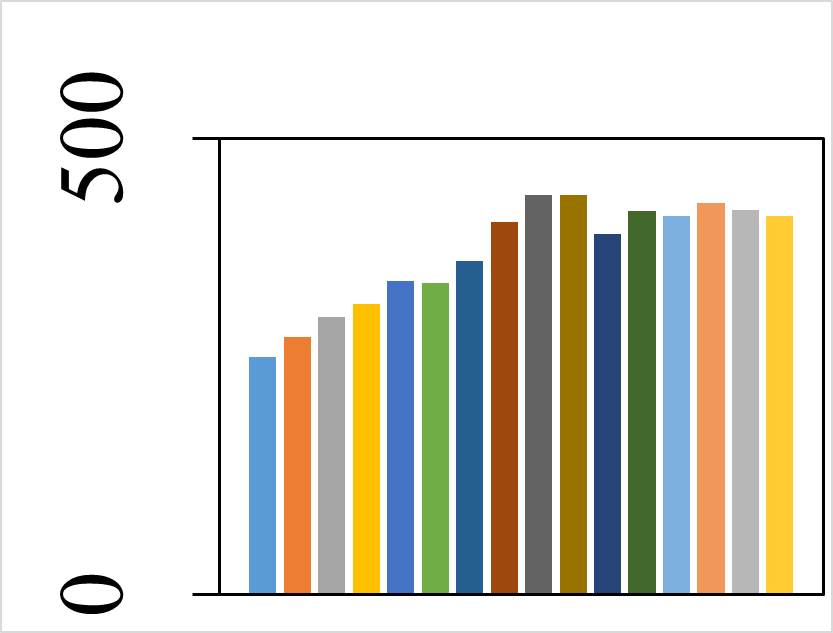

Nº residents (x10^3^)


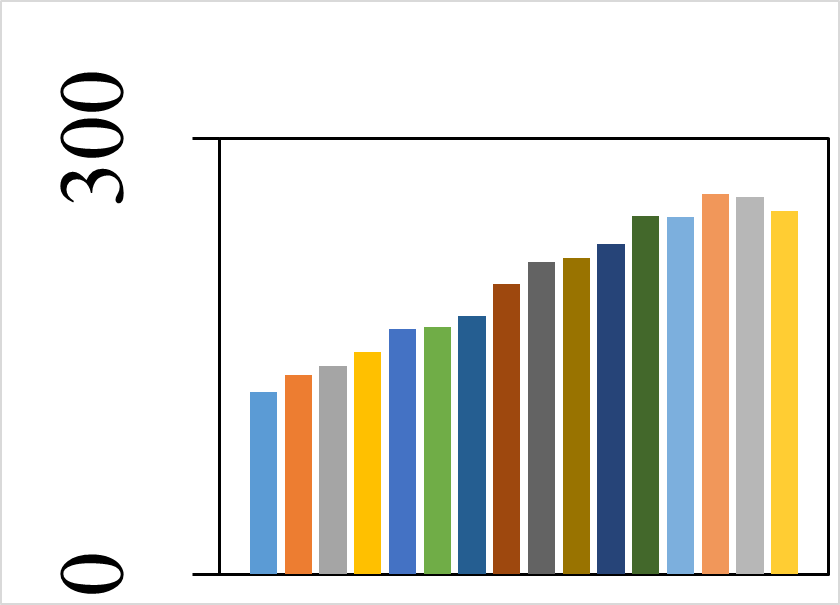


Nº residents (x10^3^)

**Fig. S2** Number of residents (thousands) in low and upper Mondego

**Figure S3**


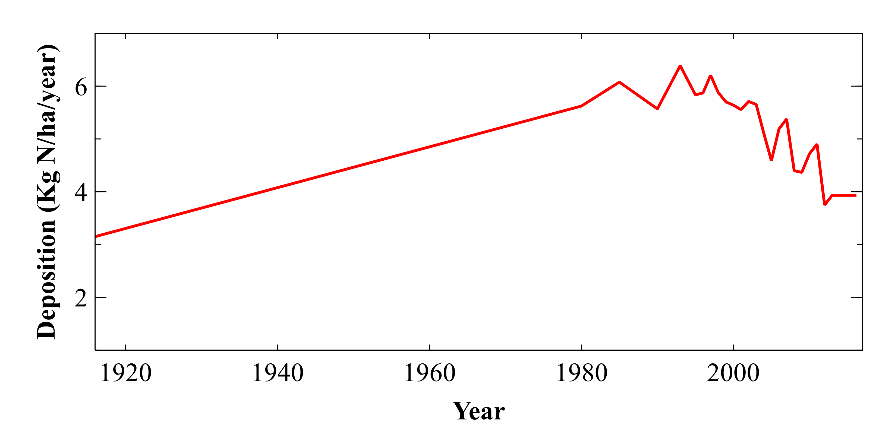


**Fig. S3** Atmospheric N deposition in the Mondego basin

**Table S1.** Areas in (ha) of the different crops in the sub-basins of upper and lower Mondego (periods 1989 to 2016)

| year | Basin | Maize | Vineyards | Olive trees | Rice | Oat | Potatoes | Wheat | All forage crops | All fruit trees | Other cultures |
| --- | --- | --- | --- | --- | --- | --- | --- | --- | --- | --- | --- |
| 1989 | Low | 18513 | 11328 | 19427 | 3238 | 2490 | 11256 | 2974 | 31221 | 7557 | 23046 |
|  | Upper | 12056 | 3989 | 6392 | 7209 | 2365 | 2166 | 952 | 12335 | 967 | 4794 |
| 1999 | Low | 11351 | 11012 | 16612 | 2435 | 1641 | 4928 | 701 | 26403 | 6871 | 9005 |
|  | Upper | 11169 | 4510 | 4150 | 5544 | 1416 | 960 | 534 | 9992 | 582 | 2214 |
| 2009 | Low | 6898 | 10566 | 14268 | 2561 | 829 | 1214 | 474 | 17158 | 6033 | 3547 |
|  | Upper | 8105 | 3045 | 2255 | 5934 | 633 | 351 | 634 | 5887 | 238 | 1432 |
| 2016 | Low | 6042 | 8944 | 10382 | 2463 | 800 | 736 | 323 | 17999 | 14764 | 4126 |
|  | Upper | 7099 | 2578 | 1648 | 5707 | 611 | 212 | 432 | 6732 | 204 | 1797 |

**Table S2.** Amount of fertilizer use per crop area and per year obtained directly from the EC (European Commission), at the Joint Research Centre of the European Union directory (<https://water.jrc.ec.europa.eu/>)

| Year | Inorganic fertilizer  (kg N/ha per crop area) |
| --- | --- |
| 1995 | 28.6 |
| 1996 | 26.3 |
| 1997 | 28.6 |
| 1998 | 26.7 |
| 1999 | 24.3 |
| 2000 | 23.4 |
| 2001 | 20.6 |
| 2002 | 24 |
| 2003 | 23.4 |
| 2004 | 23.1 |
| 2005 | 13.5 |
| 2006 | 18.8 |
| 2007 | 17.7 |
| 2008 | 19.9 |
| 2009 | 12.2 |
| 2010 | 17.1 |
| 2011 | 21 |
| 2012 | 19.7 |
